# Rethinking Colour Constancy

**DOI:** 10.1371/journal.pone.0135029

**Published:** 2015-09-10

**Authors:** Alexander D. Logvinenko, Brian Funt, Hamidreza Mirzaei, Rumi Tokunaga

**Affiliations:** 1 Glasgow Caledonian University, Glasgow, United Kingdom; 2 School of Computing Science, Simon Fraser University, Vancouver, Canada; 3 The Ritsumeikan Global Innovation Research Organization, Ritsumeikan University, Kyoto, Japan; University of Sussex, UNITED KINGDOM

## Abstract

Colour constancy needs to be reconsidered in light of the limits imposed by metamer mismatching. Metamer mismatching refers to the fact that two objects reflecting metameric light under one illumination may reflect non-metameric light under a second; so two objects appearing as having the same colour under one illuminant can appear as having different colours under a second. Yet since Helmholtz, object colour has generally been believed to remain relatively constant. The deviations from colour constancy registered in experiments are usually thought to be small enough that they do not contradict the notion of colour constancy. However, it is important to determine how the deviations from colour constancy relate to the limits metamer mismatching imposes on constancy. Hence, we calculated metamer mismatching’s effect for the 20 Munsell papers and 8 pairs of illuminants employed in the colour constancy study by Logvinenko and Tokunaga and found it to be so extensive that the two notions—metamer mismatching and colour constancy—must be mutually exclusive. In particular, the notion of colour constancy leads to some paradoxical phenomena such as the possibility of 20 objects having the same colour under chromatic light dispersing into a hue circle of colours under neutral light. Thus, colour constancy refers to a phenomenon, which because of metamer mismatching, simply cannot exist. Moreover, it obscures the really important visual phenomenon; namely, the alteration of object colours induced by illumination change. We show that colour is not an independent, intrinsic attribute of an object, but rather an attribute of an object/light pair, and then define a concept of material colour in terms of equivalence classes of such object/light pairs. We suggest that studying the shift in material colour under a change in illuminant will be more fruitful than pursuing colour constancy’s false premise that colour is an intrinsic attribute of an object.

## Introduction

Colour constancy has received a lot of attention from both psychologists [1–5], neuroscientists [6–8] and computer scientists [9–12]. Yet, there is no consensus on either what colour constancy is or the problem it presents for colour science. The phenomenon of colour constancy is usually understood as constancy of the colour of an object despite a change in the illumination [13]. As a textbook example, a ripe lemon tends to look yellow under any phase of daylight. This apparent independence of object colour from the illumination was taken for granted for a long time [13]; however, the very first experimental measurements of colour constancy yielded surprising results—colour constancy was found to be imperfect [1]. The deviations from colour constancy found in the numerous subsequent experimental studies (for a review see [6]) were sometimes so large that some have concluded that colour constancy does not exist at all [14, 15]. Nonetheless, a widely-accepted stance is: Although colour constancy is imperfect, the violation of colour constancy observed in experiments is, in general, not so serious that the very concept of colour constancy need be abandoned [5, 16–18]. We argue, however, that it in fact does need to be abandoned.

The problem arising from the colour constancy phenomenon is commonly reduced to the computational problem of illuminant estimation followed by an adjustment of the colours based on the estimated colour of the illuminant. This formulation was first put forth by Helmholtz (1867), and while it has been elaborated upon since, it has not been changed much [9–11, 19]. When the illumination incident upon an object changes, the light reflected from the object changes, as do the cone excitations induced by the reflected light. The Helmholzian idea was that once one knows the illuminant change (represented in terms of the change in cone excitations in response to an ideal reflector) it can be taken into account so as to compensate for the corresponding change in the other cone excitations. Accounting for the illumination in this way is often referred to as “discounting” the illumination [20], although generally the information about the illuminant only needs to be separated out, it does not need to be completely discarded.

It should be noted that most computational studies of colour constancy, whether based on estimating the illuminant or otherwise, implicitly assume perfect colour constancy as their goal. However, as mentioned before, colour constancy has never been found to be perfect. Is this imperfectness because of the imperfectness of illuminant estimation or is there perhaps some other fundamental underlying problem? Many in the colour science community believe that given a perfect estimate of the illuminant, reasonably good, if not perfect, colour constancy will follow, which is one reason why so much energy has been expended by colour scientists in an effort to solve the illumination estimation problem [21]. Note that we are not concerned here with illumination estimation as a source of colour inconstancy—we will assume it is perfect—and then consider the other possible causes of inconstancy.

Since the only thing to which the brain has access is generally believed to be the cone excitations (and/or some other factors such as those resulting from early neuronal processing) produced by the illuminant spectral power distribution times the object spectral reflectance, numerous computational algorithms have been proposed for estimating the tristimulus values of the illuminant [21, 22]. Less effort has been devoted to the problem of what use can be made of the cone responses to the illuminant if they are available. Generally, the approach has been to use them to derive a linear—usually diagonal [23–27]—transformation of the cone excitation space that is expected to model the effect of the illuminant on the cone excitations. More specifically, the cone excitations obtained from an object lit by daylight, or some other “canonical” light, are taken to represent the “true” colour of the object. Given the cone excitations induced by the illuminant, the derived transformation is then used to transform the cone responses arising from the light reflected from an object to the cone responses arising from the light that would be reflected by the object if it were to be lit instead by daylight. We will refer to the problem of deriving the cone excitations (or XYZ tristimulus values) under the canonical illuminant given only the cone excitations (or XYZ tristimulus values) arising from a scene under an unknown illuminant as the *computational colour constancy problem* [10].

However, as is well known the computational colour constancy problem is ill-posed. Many people in the computational colour constancy field view the problem as being ill-posed in the sense of being one having too many unknowns with too few constraints [21]. The focus, then, is to determine more constraints, as for example, by assuming the average colour is grey [28] or that the highest value in a colour channel represents 100% reflectance in that channel [12, 29], or that the reflectances lie in a low-dimensional subspace [30]. However, unless reflectances are restricted to a 3-parameter family the problem is ill-posed in a deeper sense [31]; namely, there is no solution to it at all.

There being no solution stems from metamer mismatching. Metamer mismatching is usually described as the fact that two objects producing the same cone responses under one illuminant will generally produce different cone responses under another illuminant [32, 33]. As a consequence of metamer mismatching, any transformation of the cone excitations intended to account for the change in illuminant that brings about colour constancy for one object will generally fail to do so for many others. For these objects, instead of colour constancy, colour inconstancy will be observed. The degree of such inconstancy will depend on the extent of metamer mismatching, which can be measured in terms of the metamer mismatch index proposed by Logvinenko et al. [34]. Furthermore, this inconstancy can be quantified in terms of so-called material colour shift, which is mainly determined by metamer mismatching [34]. One of the main objectives of the present paper is to compare the extent of metamer mismatching for the surfaces and illuminants used in the previous colour constancy study by Logvinenko & Tokunaga [35] to the degree of colour inconstancy they uncovered in their experiments.

A second objective is to develop an alternative to the existing colour constancy framework. In many ways the existing futile search for colour constancy is similar to the search for aether as a medium for the transmission of light waves. The existence of aether was an intuitively reasonable, but incorrect hypothesis, with the result that much effort was wasted trying to establish it. In place of searching for colour constancy, we propose further development of a new theoretical framework [36] involving asymmetric colour matching functions and a formal definition of material colour as an equivalence class of object/light pairs; and suggest that rather than continuing to debate the colour constancy problem, the focus be placed instead on determining the asymmetric colour matching functions and evaluating the degree of material colour shift.

## Metamer Mismatching: Theory

In this section we will briefly introduce the terminology and notation (also see the glossary of terms in S1 Appendix) needed to expose the main notions concerning metamer mismatching, which are presented at length elsewhere [34]. While being rather technical, these notions are much needed since it is in terms of them that the role of metamer mismatching (which we believe has been underestimated by far) in the colour constancy issue will be shown. Consider a set of colour mechanisms (e.g., cone photoreceptors or camera sensors) Φ = (*φ*_1_, *φ*_2_, *φ*_3_), the response of each of which to a reflecting object with spectral reflectance function *x*(*λ*) illuminated by a light with spectral power distribution *p*(*λ*) is given by

$$\begin{array}{c} \varphi_{i}(x)=\int_{\lambda_{\min}}^{\lambda_{\max}}x(\lambda )p(\lambda )s_{i}(\lambda )d\lambda \;(i=1,2,3), \end{array}$$ (1)

where [*λ*_min_, *λ*_max_] is the visible spectrum interval, and *s*_*i*_(*λ*) is the spectral sensitivity of the *i* ^*th*^ colour mechanism. We consider only non-specular, matte surfaces. The vector Φ(*x*) = (*φ*_1_(*x*), *φ*_2_(*x*), *φ*_3_(*x*)) of the colour mechanism responses will be referred to as the *colour signal* produced by the colour mechanism set Φ in response to *x*(*λ*) illuminated by *p*(*λ*). Let 𝓧 denote the set of all spectral reflectance functions (i.e., X = {0 ≤ *x*(*λ*) ≤ 1}). As *x*(*λ*) is run over 𝓧, the set of corresponding colour signals, Φ(𝓧), forms a convex volume in the colour signal (cone excitation) space, which is conventionally referred to as the *object-colour solid* [33]. The shape of the object-colour solid clearly depends on the illuminant *p*(*λ*). We will use subscripts to indicate the illuminant under which a particular object-colour solid is produced. For example, denoting the canonical illuminant as *p*_0_(*λ*), the object-colour solid for this illuminant will be denoted as Φ_*p*_0__(𝓧). It will be referred to as the *canonical object-colour solid*.

Given some other illuminant, *p*(*λ*), consider the object-colour solids Φ_*p*_0__(𝓧) and Φ_*p*_(𝓧). Each spectral reflectance function *x*(*λ*) maps to a point in each object-colour solid: Φ_*p*_0__(*x*) in the canonical object-colour solid, and Φ_*p*_(*x*) in Φ_*p*_(𝓧). Let us establish a correspondence (denoted as *ρ*) between the object-colour solids Φ_*p*_0__(𝓧) and Φ_*p*_(𝓧) that associates with one another the points in these two object-colour solids that are produced by a common spectral reflectance function. Specifically, two points *z*′ ∈ Φ_*p*_0__(𝓧) and *z*′′ ∈ Φ_*p*_(𝓧) are, by definition, in *ρ*-correspondence (written as *z*′*ρz*′′) if and only if there is a spectral reflectance function *x*(*λ*) such that *z*′ = Φ_*p*_0__(*x*) and *z*′′ = Φ_*p*_(*x*). The belief (descending from Helmholtz) is that, given a point *z*′′ in the object-colour solid Φ_*p*_(𝓧) produced by a spectral reflectance function *x*(*λ*), the inverse *ρ*-image of it (i.e., the point *z*′ = Φ_*p*_0__(*x*) in the canonical object-colour solid Φ_*p*_0__(𝓧), produced by spectral reflectance function *x*(*λ*)) can be used to represent the “true” colour of the object having spectral reflectance *x*(*λ*).

The problem is that if the correspondence *ρ* is thought of as a map, then it should be recognized as being a multivalued map because a single point *z* in the canonical object-colour solid Φ_*p*_0__(*x*) turns out to be in *ρ*-correspondence with a whole set, denoted as *ρ*(*z*; *p*_0_, *p*), of points in the object-colour solid Φ_*p*_(*x*). This can be expressed formally as

$$\begin{array}{c} \rho (z;p_{0},p)=\underset{x\in \Phi_{p_{0}}^{-1}\left(z\right)}{⋃}\Phi_{p}(x)\text{,} \end{array}$$ (2)

where $\Phi_{p_{0}}^{-1}\left(z\right)$ is the set of spectral reflectance functions mapping to *z*, i.e., $\Phi_{p_{0}}^{-1}\left(z\right)=\{x\in X:\Phi_{p_{0}}(x)=z\}$.

**Fig 1:**
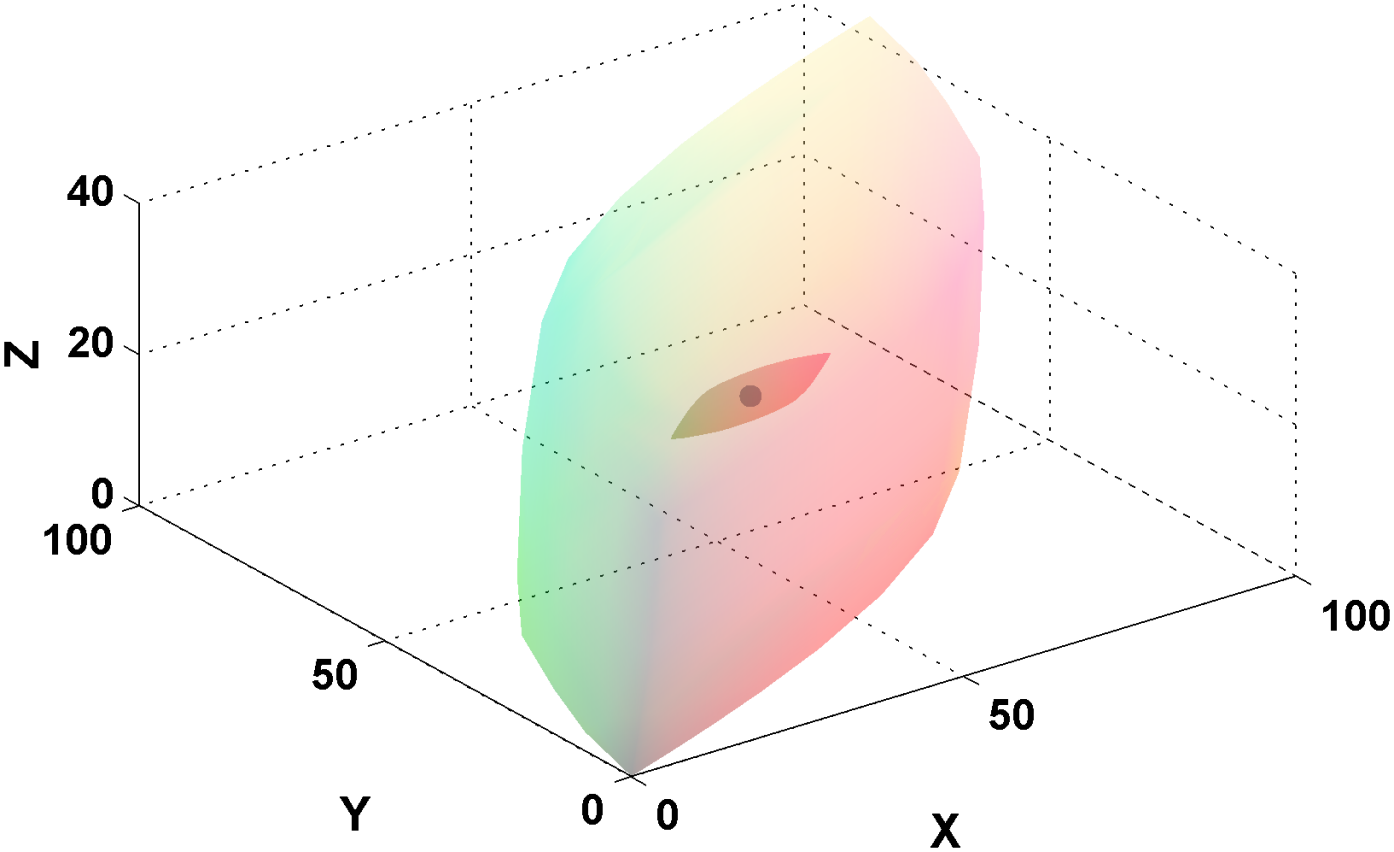
Metamer mismatch volume for the flat grey spectral reflectance function (see text) when the illuminant changes from CIE D65 to CIE A is shown inside the object-colour solid for CIE A. The coordinates are XYZ in the CIE 1931 colorimetric space. The grey dot indicates the location of the colour signal corresponding to the flat grey reflectance. It is located at the centre of the object colour solid and lies inside the metamer mismatch volume.

For every interior point *z* ∈ Φ_*p*_0__(𝓧), *ρ*(*z*; *p*_0_, *p*) is a convex body in Φ_*p*_(𝓧), which will be referred to as the *metamer mismatch volume* induced by the shift from the illuminant *p*_0_ to the illuminant *p*. Fig 1 shows an example of a metamer mismatch volume computed using the algorithm described elsewhere [34] for a point produced by the flat spectral reflectance function taking 0.5 across the whole visible spectrum interval, i.e., *x*(*λ*) = 0.5 for each *λ*. This reflectance will be referred to as *flat grey*. The CIE 1931 colour matching functions [33] were used as the *s*_*i*_(*λ*) in Eq (1). CIE illuminant D65 was taken as the canonical illuminant *p*_0_(*λ*), and CIE illuminant A was used for *p*(*λ*).

Similar to Eq (2), for a single point *z* in the object-colour solid Φ_*p*_(*x*) there is an entire subset of points in the canonical object-colour solid Φ_*p*_0__(*x*) that are in *ρ*-correspondence with *z* (written as *ρ* ^−1^(*z*; *p*_0_, *p*)):

$$\begin{array}{c} \rho^{-1}(z;p_{0},p)=\underset{x\in \Phi_{p}^{-1}\left(z\right)}{⋃}\Phi_{p_{0}}(x)\text{,} \end{array}$$ (3)

where $\Phi_{p}^{-1}\left(z\right)=\{x\in X:\Phi_{p}(x)=z\}$.

By analogy with ordinary functions, Eq (3) can be treated as the inverse image of *z* = Φ_*p*_(*x*). Since *ρ* ^−1^(Φ_*p*_(*x*);*p*_0_, *p*) is a whole volume rather than a singleton, there is a great deal of uncertainty concerning which of the points in *ρ* ^−1^(Φ_*p*_(*x*);*p*_0_, *p*) should be taken as a descriptor of the “true” colour. This presents the dilemma that if an object under one light can become any one of a wide range of colours under a second light, which is to be considered its true colour? Since there is no unique answer, this means that the computational colour constancy problem is ill-posed even if we know the full spectra of both illuminants *p* and *p*_0_, let alone when the illuminant *p* is unknown and has to be estimated.

Interestingly, the computational colour constancy problem becomes well-defined for the restricted case of reflectances that map to the object-colour solid boundaries. Indeed, there is no metamerism on the object-colour solid boundaries [37], so there is no metamer mismatching there either. As a result, for points on the object-colour solid boundaries (written as ∂Φ_*p*_0__(𝓧) and ∂Φ_*p*_(𝓧)), *ρ* is a one-to-one map: ∂Φ_*p*_0__(𝓧) → ∂Φ_*p*_(𝓧). Therefore, given *z* ∈ ∂Φ_*p*_(𝓧), there exists a unique inverse *ρ* ^−1^(*z*; *p*_0_, *p*) that is a unique point, $\Phi_{p_{0}}(\Phi_{p}^{-1}\left(z\right))$, on the boundary of the canonical object-colour solid Φ_*p*_0__(𝓧). In other words, *ρ* ^−1^(*z*; *p*_0_, *p*) is a singleton for any *z* lying on the boundary surface of the object-colour solid Φ_*p*_(𝓧) that can be expressed as

$$\begin{array}{c} \rho^{-1}(z;p_{0},p)=\Phi_{p_{0}}(\Phi_{p}^{-1}\left(z\right)). \end{array}$$ (4)

Note that while Eq (4) does define a one-to-one map between ∂Φ_*p*_(𝓧) and ∂Φ_*p*_0__(𝓧), this map is non-linear. Hence, any attempt to use a linear transformation—much less a diagonal one such as the von Kries transformation—to model this map cannot succeed in principle even for points on the object-colour solid boundary, let alone for the whole cone excitation space as is required in the computational approach to colour constancy.

An alternative approach might be to seek an appropriate map as an approximation of the correspondence *ρ*, the rationale being that such an approximating map might achieve “approximate” colour constancy. The idea is that, although it is still not clear which of the points in *ρ* ^−1^(*z*; *p*, *p*_0_) should be taken as an estimate of the “true” colour of the object, one can approximate the whole volume *ρ*(*z*; *p*, *p*_0_) by a point in it such as its centroid. However, this approach makes sense only if the mismatching of metamers is not large. A commonly held view is that the effect of metamer mismatching is, generally, so small that it can simply be assumed to be insignificant; however, we show below that the amount of metamer mismatching is too large to ignore.

## Metamer mismatch volumes for 4 illumination conditions

In order to look into the implications of metamer mismatching for colour constancy, we computed the metamer mismatch volumes for the colour stimuli of Logvinenko & Tokunaga [35]. These stimuli are particularly useful for our analysis of metamer mismatching because they form a set of well-defined (i.e., Munsell standard) coloured papers that evenly represents all the object colour hues, and for which asymmetric colour matching data for a large set of illumination conditions (36 pairs of illuminants) is also available.

As the metamer mismatch volume described by Eq (2) is a convex body, it is fully determined by its boundary surface. These boundaries were evaluated by using the recent algorithm [34] that generates 5-transition reflectances that map either directly to the metamer mismatch boundary or deviate from it only slightly. For each metamer mismatch body evaluated in this paper, 1000 such 5-transition reflectances have been produced. All these reflectances have been checked to ensure that they are, in fact, metameric under the first illuminant. A complete database of the 5-transition reflectance functions for all the metamer mismatch volumes are available in S1 Dataset. Anyone who wishes can easily verify that the volumes are correct by computing the XYZ tristimulus values of the reflectances under the specified lights and then confirming that they are all of equal XYZ under the first light, yet describe a volume under the second light.

**Fig 2:**
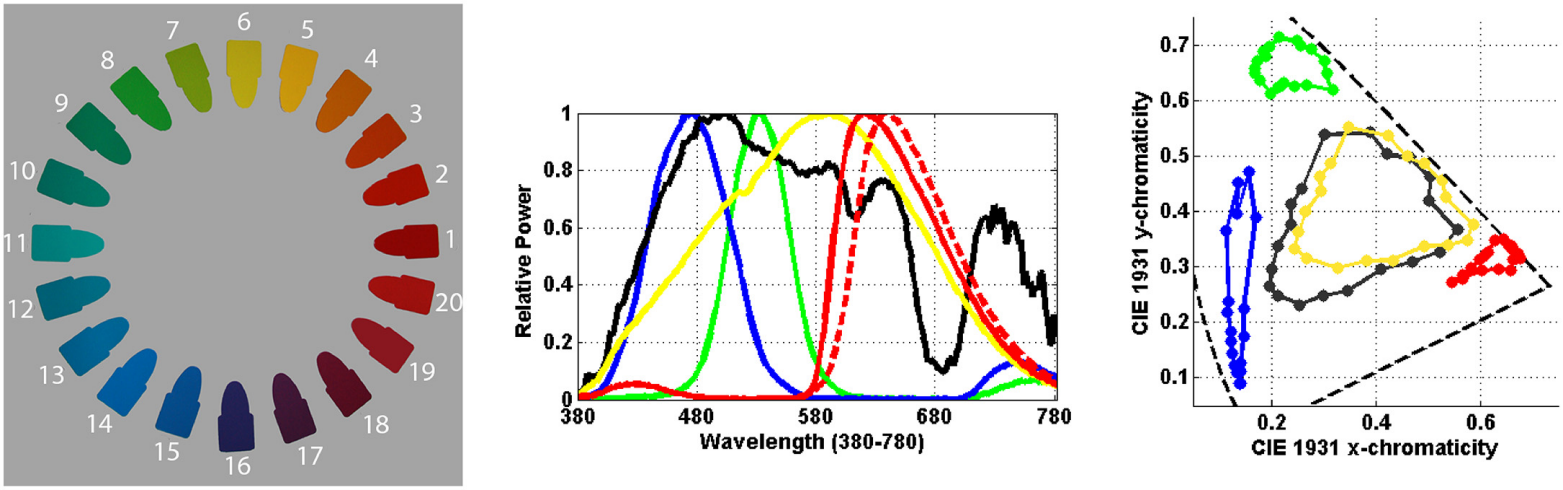
(Left) Photograph giving a general indication of the colours of the 20 chromatic stimulus papers used in Logvinenko & Tokunaga’s experiment. To evaluate the colours correctly requires viewing the actual Munsell papers. Their Munsell notations starting from 1 are: 10RP 5/14, 5R 4/14, 10R 5/16, 5YR 7/14, 10YR 7/14, 5Y 8/14, 10Y 8.5/12, 5GY 7/12, 10GY 6/12, 5G 5/10, 10G 5/10, 5BG 6/10, 10BG 5/10, 5B 5/10, 10B 5/12, 5PB 5/12, 10PB 4/12, 5P 4/12, 10P 4/12, 5RP 5/12. (Centre) Spectral power distribution of the illuminants employed in Logvinenko & Tokunaga’s experiment. The line colour indicates the colour of the light each line represents. The R2 light is represented by the dashed red line and the neutral light by the black line. (Right) The dots indicate the CIE 1931 chromaticity coordinates of the light reflected from the 20 stimulus papers under all the lights except R2. The marker colour indicates the colour of the corresponding light.

Logvinenko & Tokunaga used the 20 chromatic Munsell papers [38] depicted in Fig 2 (left) along with a grey (N5/) and a black (N1/) paper. Six different lights were used to illuminate the papers: neutral (N), yellow (Y), blue (B), green (G), and two reds (R1 and R2). Their spectral power distributions are plotted in Fig 2 (centre). Fig 2 (right) presents the CIE 1931 chromaticity coordinates of the stimulus papers under five of the lights. Results for R1 and R2 were found to be rather similar, so in what follows R will be used to denote R1, while R2 is excluded from further consideration.

**Fig 3:**
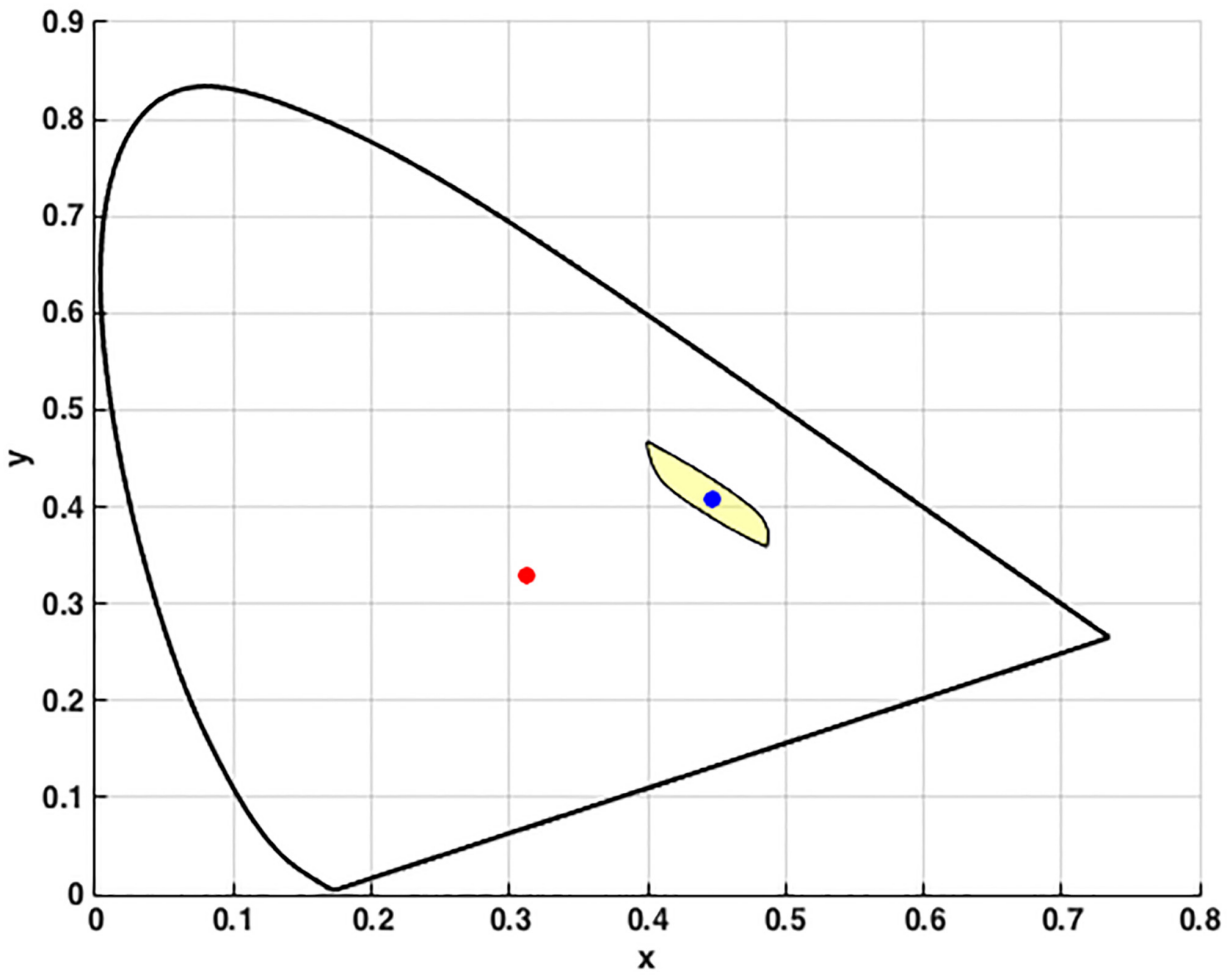
Chromaticity mismatch area (yellow area) for the flat grey reflectance when CIE illuminant D65 is replaced by CIE illuminant A plotted in the CIE 1931 xy-chromaticity diagram. The red dot indicates the chromaticity of the flat grey under CIE D65 and the blue dot the corresponding chromaticity under CIE A lying inside the metamer mismatch area.

We evaluated the metamer mismatch boundary surfaces for the chromatic Munsell papers depicted in Fig 2 (left) under all 8 pairs of illuminants that include the neutral. A pair of illuminants, for example N and Y, will be referred to as an illumination condition and written as NY. All computation was done using the CIE 1931 colour matching functions [33] as *s*_*i*_(*λ*) in Eq (1). Since the overlap between multiple mismatch volumes can be hard to see in a 3D plot, we will instead plot 2D projections of the volumes in the CIE 1931 chromaticity diagram. Specifically, the set of the *xy* chromaticity coordinates of the points in a metamer mismatch volume defines a two-dimensional area in the CIE chromaticity plane showing how the initial chromaticity is dispersed due to metamer mismatching. We will refer to such an area as the *chromaticity mismatch area*. Fig 3 depicts the chromaticity mismatch area corresponding to the metamer mismatch volume for the flat grey from Fig 1.

**Fig 4:**
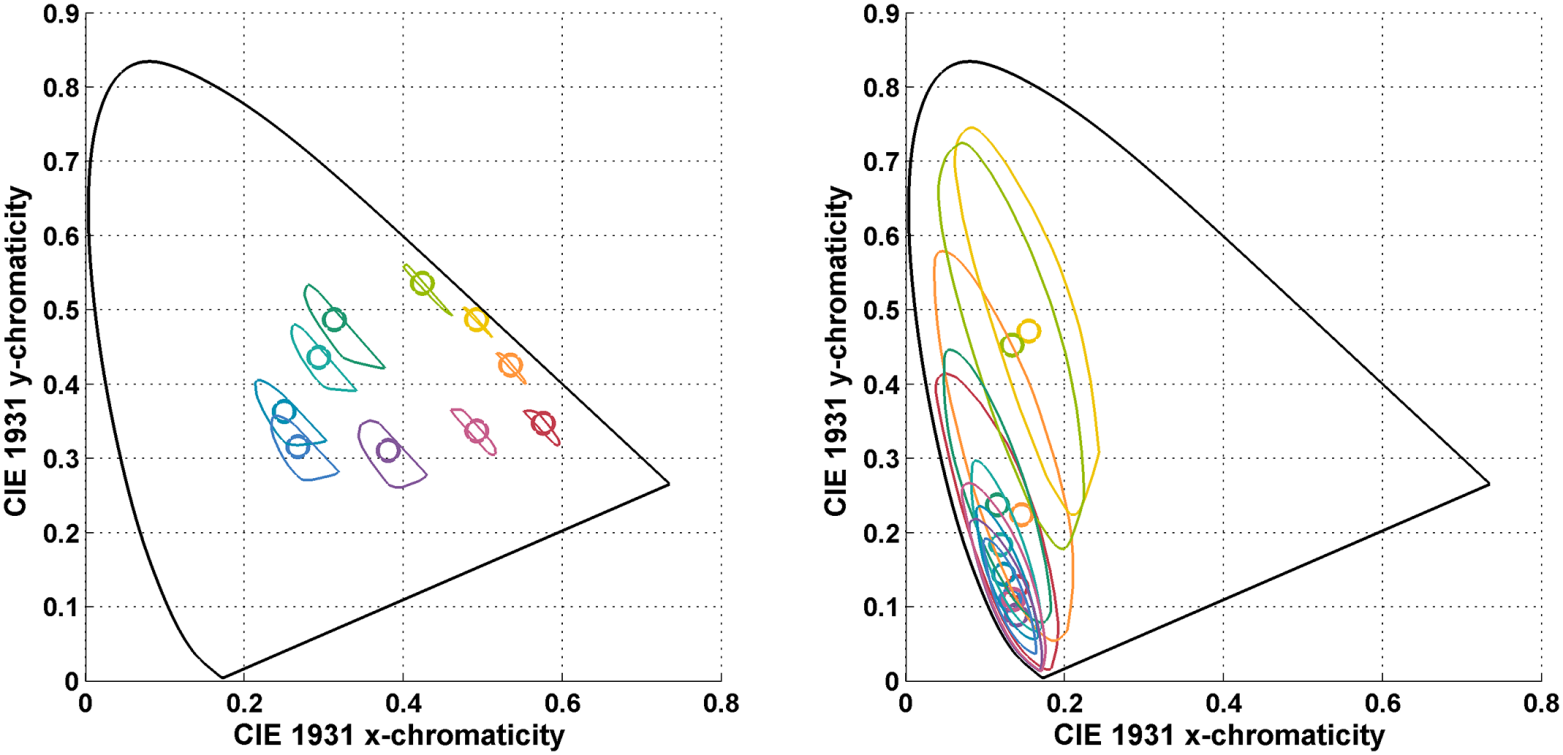
(Left) The chromaticity mismatch area for a shift from the neutral (N) illuminant to the yellow (Y) illuminant. (Right) The chromaticity mismatch area for a shift from the neutral to the blue (B) illuminant. Plots are in the CIE 1931 xy-chromaticity diagram. The circles are the chromaticities of the 10 odd-numbered Munsell papers from Fig 2 (left) under the second illuminant in each case. The closed contours indicate the boundaries of the metamer mismatch areas. The colour of the circles and boundaries of the metamer mismatch areas correspond to one another, and very roughly indicate the colours of the Munsell papers.

**Fig 5:**
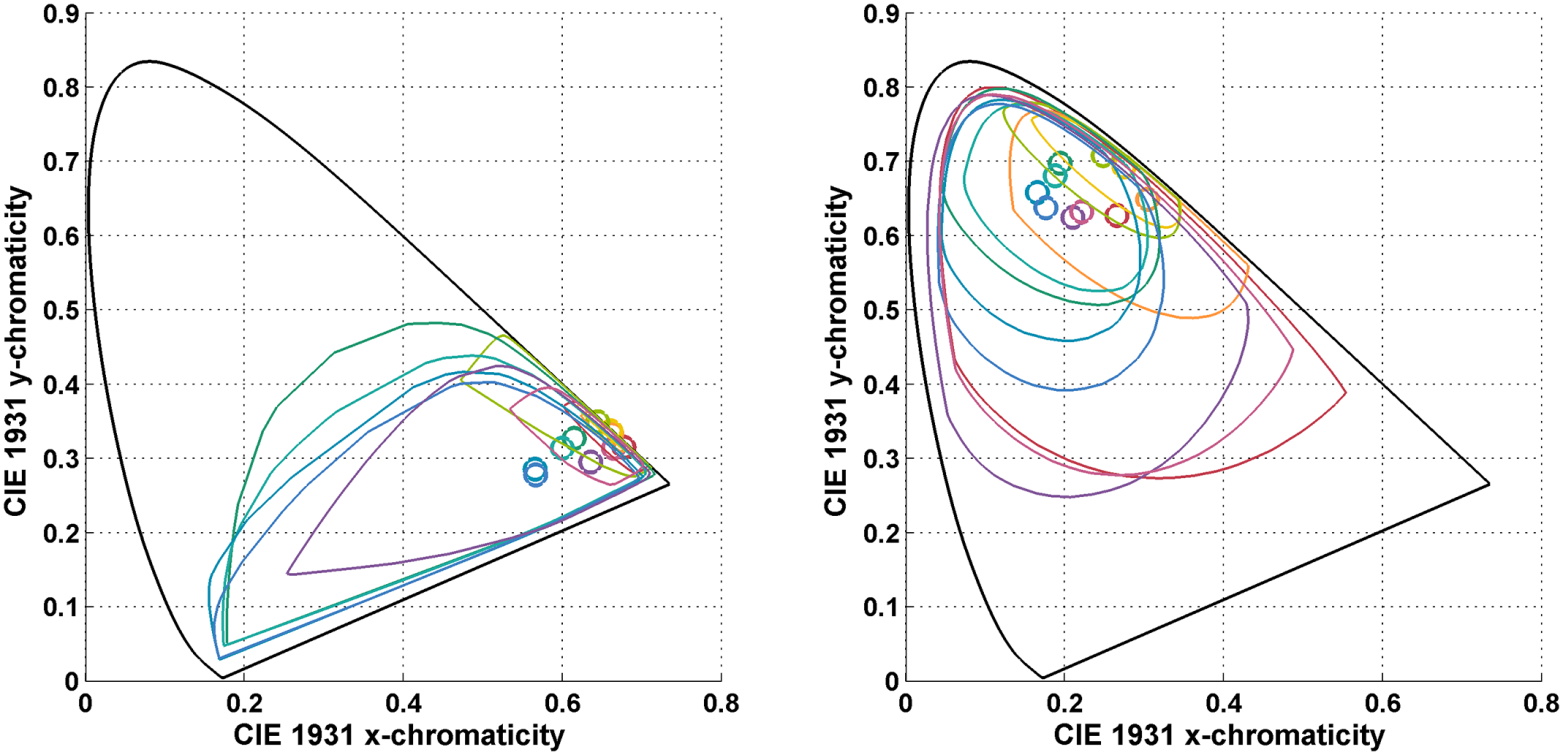
(Left) The chromaticity mismatch area for a shift from the neutral (N) to the red (R) illuminant. (Right) The chromaticity mismatch area for a shift from the neutral (N) to the green (G) illuminant. Plots are in the CIE 1931 xy-chromaticity diagram. The circles are the chromaticities of the 10 odd-numbered Munsell papers from Fig 2 (left) under the second illuminant in each case. The closed contours indicate the boundaries of the metamer mismatch areas. The colour of the circles and boundaries of the metamer mismatch areas correspond to one another, and very roughly indicate the colours of the Munsell papers.

Fig 4 shows the chromaticity mismatch areas resulting from shifts from the neutral illuminant to the yellow and to the blue illuminants (i.e., for the NY and NB illumination conditions), and Fig 5 for the shift to the red and green illuminants (i.e., for the NR and NG illumination conditions). In other words, the chromaticity mismatch areas were computed for a representative sample of the coloured points in Fig 2 (right) for each of these four illumination conditions. Clearly, the chromaticity mismatch areas are large, even for the NY illumination condition.

In order to evaluate metamer mismatching quantitatively we computed the *metamer mismatch index* as suggested by Logvinenko et al. [34]. Specifically, they define the metamer mismatch index, *i*_*mm*_(*z*; *p*_1_, *p*_2_), as the ratio of the metamer mismatch volume to that of the object colour solid. In particular, for a point *z* in Φ_*p*_1__(𝓧) under illuminant *p*_1_ that under illuminant *p*_2_ disperses into a metamer mismatch volume *ρ*(*z*; *p*_1_, *p*_2_) in Φ_*p*_2__(𝓧), the metamer mismatch index is:

$$\begin{array}{c} i_{mm}(z;p_{1},p_{2})=\frac{v(\rho (z;p_{1},p_{2}))}{v(\Phi_{p_{2}}(X))}, \end{array}$$ (5)

where *v*(*ρ*(*z*; *p*_1_, *p*_2_)) is the volume of *ρ*(*z*; *p*_1_, *p*_2_), and *v*(Φ_*p*_2__(𝓧)) is the volume of the Φ_*p*_2__(𝓧) object-colour solid.

**Fig 6:**
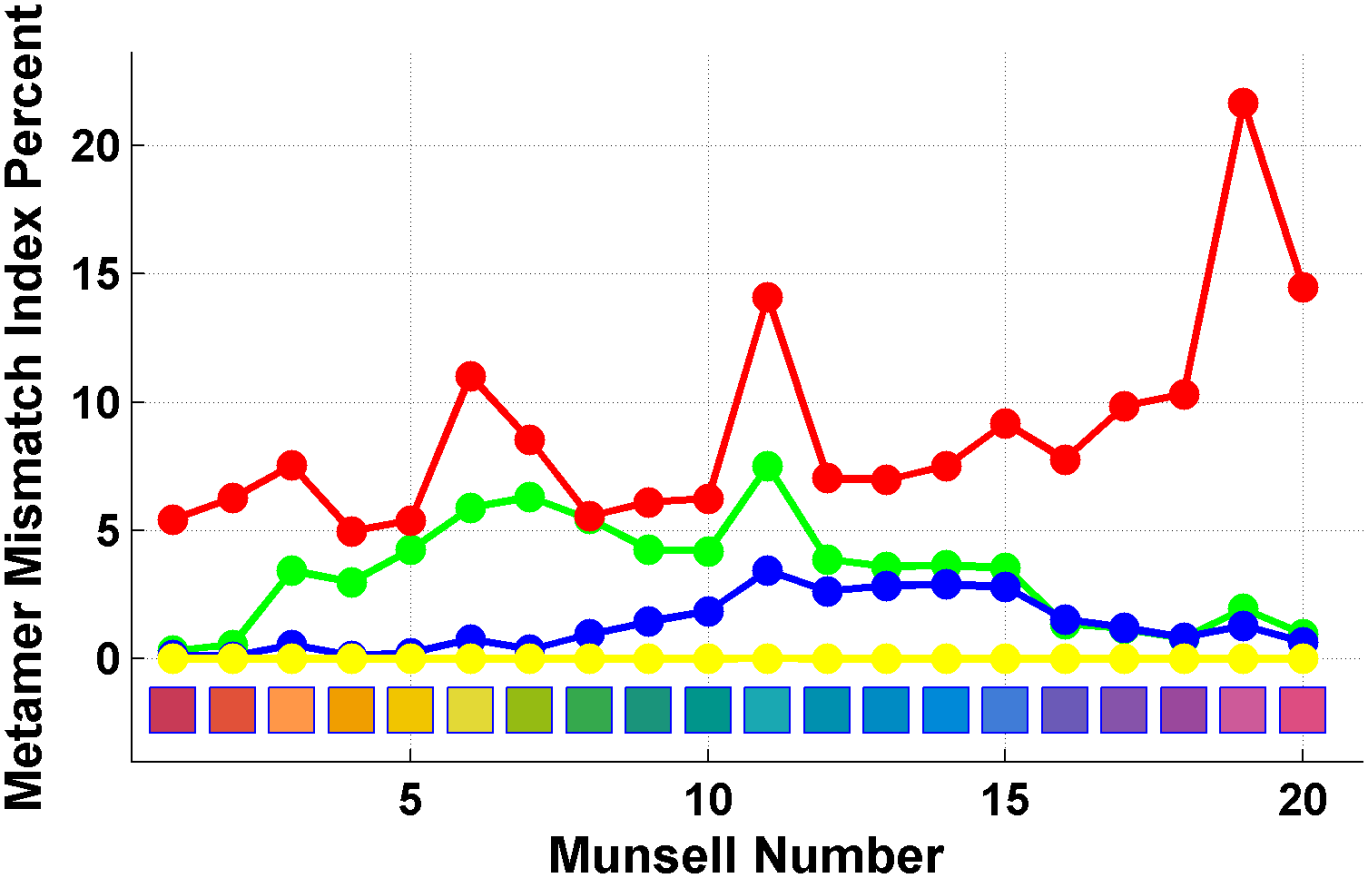
Metamer mismatch indices in percent (from Table 1) for the shift from the neutral to a coloured illuminant. The marker colour indicates the colour of illuminant. The abscissa is the number of the corresponding Munsell paper from Fig 2 (left).

The metamer mismatch indices in percent for 8 illumination conditions and 20 Munsell papers plus flat grey are presented in Table 1 and Fig 6. For example, the first entry—0.32%—stands for *i*_*mm*_(*z*_1_; *N*, *G*), where *z*_1_ is the CIE tristimulus triple for Munsell paper #1 (Fig 2 (left)) as evaluated for the neutral (N) illuminant.

**Table 1:** Metamer-mismatch indices in percent. Rows correspond to papers that are numbered as in Fig 2 (left). Columns correspond to illumination conditions.

Munsell Paper	Illumination Condition							
	NG	NB	NY	NR	GN	GB	GY	GR
1	0.32	0.14	0.0024	5.4	1.1	0.69	0.0023	9.3
2	0.55	0.10	0.0019	6.3	1.2	0.53	0.0019	7.2
3	3.4	0.55	0.0064	7.5	4.1	1.3	0.0059	9.1
4	3.0	0.13	0.0047	5.0	2.3	0.54	0.0026	5.5
5	4.2	0.23	0.0048	5.4	3.9	0.70	0.0042	5.9
6	5.9	0.77	0.0088	11	5.7	1.4	0.0082	9.6
7	6.3	0.37	0.0056	8.5	4.9	0.86	0.0051	6.6
8	5.5	0.96	0.0072	5.6	6.1	1.3	0.0068	6.9
9	4.3	1.5	0.0089	6.1	6.3	1.9	0.0086	9.8
10	4.2	1.9	0.0090	6.3	6.7	2.1	0.0097	11
11	7.5	3.4	0.016	14	8.4	2.8	0.017	14
12	3.9	2.6	0.0102	7.0	6.8	2.5	0.011	13
13	3.6	2.9	0.0105	7.0	6.7	2.7	0.011	13
14	3.7	2.9	0.0110	7.5	6.9	2.8	0.012	14
15	3.5	2.8	0.0117	9.2	6.5	3.0	0.013	15
16	1.4	1.5	0.0075	7.8	4.0	2.0	0.0083	15
17	1.2	1.2	0.0074	9.9	3.4	1.9	0.0079	15
18	0.81	0.84	0.0064	10	2.7	1.6	0.0066	14
19	2.0	1.29	0.0078	22	4.0	1.9	0.0074	16
20	0.98	0.65	0.0051	15	2.9	1.4	0.0054	13
Flat Grey	13	4.1	0.021	47	9.7	3.1	0.018	20
Average Excluding Flat Grey	3.3	1.3	0.0077	8.8	4.7	1.7	0.0077	11.1
Average Including Flat Grey	3.8	1.5	0.0083	10.6	5.0	1.8	0.0082	11.6

As might be expected, the least serious metamer mismatching is observed for the yellow illuminant. Its metamer mismatch index averaged across all papers (excluding flat grey) is just 0.008% for the YN and NY conditions. The largest amount of mismatching occurs with the red illuminant for which the average metamer mismatch index is 9% and 11% for the NR and RN conditions, respectively.

Note that by eye, the metamer mismatching in Figs 4 and 5 seems to be more pronounced than indicated by the index values found in Table 1. This impression arises in part because the metamer mismatch volumes can be thin, with less mismatching along the luminance dimension than in the chromaticity domain. It also arises because the metamer mismatch index is a measure of volume, not a linear dimension. For example a hypothetical metamer mismatch volume having the same shape as the object colour solid but 1/4 its size would have a metamer mismatch index of (1/4)^3^ × 100 = 1.6%.

The large size of the metamer mismatch volumes demonstrates that approximating the correspondence *ρ* by any (not to mention linear) map is not something that it is worthwhile to do. Consider a simple analogy from mathematical statistics involving two random variables. Clearly, there is not too much sense in regressing one random variable to the other when their scatter plot has the form of a circular, disk-like cloud. If metamer mismatching is taken as the analog of variability, one can see that a linear approximation of the correspondence *ρ* does not make much sense.

Even more important than the chromaticity mismatch area’s size is that the chromaticity mismatch area for a single paper covers the chromaticities of many other papers. For example, for the blue illuminant, the metamer mismatch area of paper #2 covers the chromaticities of 18 of the 20 Munsell papers. For the green and red illuminants, some of metamer mismatch areas cover the entire set of 20 Munsell papers. The ramifications of this are considered below in section “Colour Constancy or Colour Alteration?”

One might even expect that observers would see very little colour variation under the red, green and blue illuminants of Fig 2 (centre), but the fact is that observers report seeing a wide range of colours under all of them. Admittedly, Logvinenko & Tokunaga (2011) found some decrease in asymmetric matching performance when these three illuminants were involved, but overall their performance was considerably better than one might expect in the view of the substantial degree of metamer mismatching found in Figs 4 and 5. The problem, then, is to understand how this can happen in light of the fact that the metamer mismatch volumes are very large. In the next section we look into this issue in more detail.

## Analysis of Logvinenko & Tokunaga’s data

Logvinenko & Tokunaga performed an asymmetric colour matching experiment [35]. They analyzed the results of their experiment in various ways, but not in the context of metamer mismatching. At the time of their analysis, there did not exist an algorithm for computing the exact metamer mismatch volumes, however, one has been developed since [34], and using it we can now analyze the results of their experiment in light of metamer mismatching. In particular, our new analysis shows that their observers’ performance is much better than might be expected given the potential extent of metamer mismatching, and also much better than that obtained using a von Kries type adaptation transform. These findings motivate our suggestion to reconsider the very approach to the colour constancy issue.

**Fig 7:**
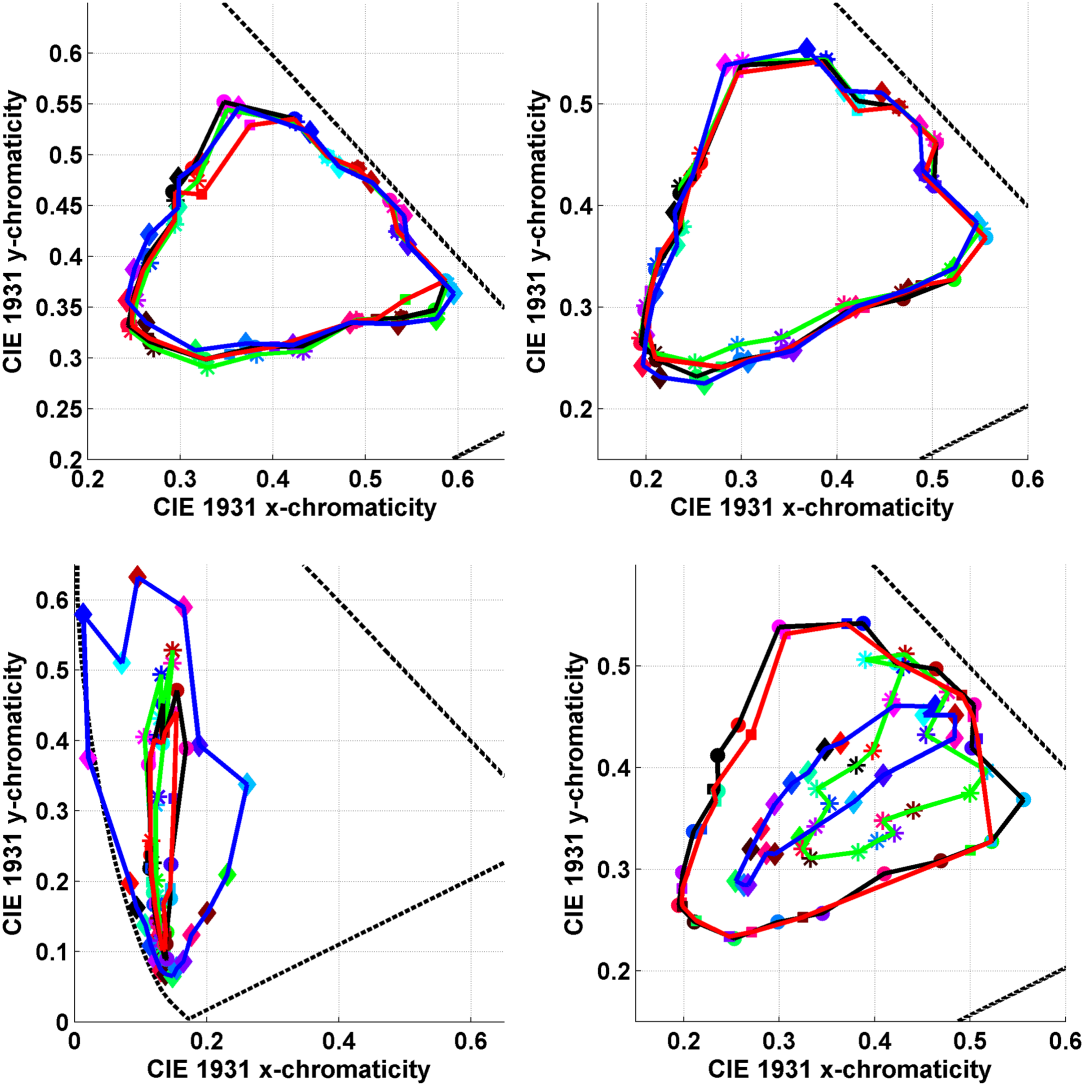
Comparison of asymmetric matches under 4 different illumination conditions (see Fig 8 for more). The asymmetric colour matches made by observers in Logvinenko & Tokunaga’s experiment are compared to predictions based on the mismatch centroid chromaticities, and on the von Kries transformation. The axes are those of the CIE 1931 xy-chromaticity diagram. Circular dots connected by the black lines stand for the stimulus papers. The squares connected by the red lines represent the averaged observer matches. Asterisks connected by the green lines stand for the mismatch centroid chromaticities. Diamonds connected by the blue lines indicate the prediction using the von Kries transformation. The dashed line is a segment of the spectrum locus. (a) Neutral/Yellow asymmetric colour matching condition. (b)Yellow/Neutral asymmetric colour matching condition. Note, however, that in (b) the squares connected by red lines indicate matches made under the neutral (N) illuminant, whereas in (a) the matches were under the yellow (Y) illuminant and so forth. (c) Blue/Neutral asymmetric colour matching condition. Note that one of the predictions using the von Kries transformation (blue curve) in fact falls outside the spectrum locus. (d) Neutral/Blue asymmetric colour matching condition.

**Fig 8:**
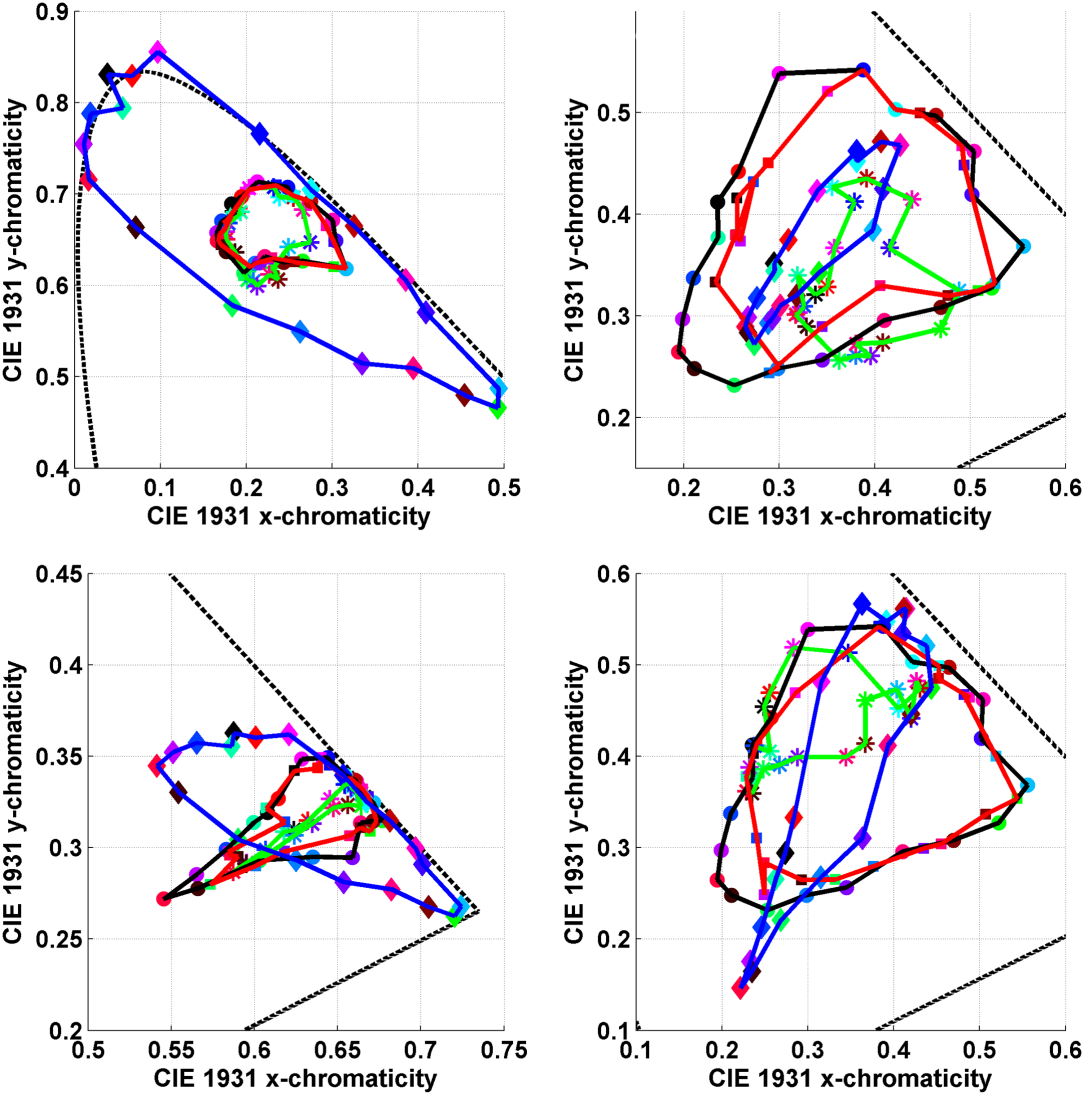
Continuation of the comparisons in Fig 7. The axes are those of the CIE 1931 xy-chromaticity diagram. (Upper Left) Neutral/Green asymmetric colour matching condition where again some of the predictions using the von Kries transformation (blue curve) fall outside the spectrum locus. (Upper Right) Green/Neutral asymmetric colour matching condition. (Lower Left) Neutral/Red asymmetric colour matching condition. (Lower Right) Red/Neutral asymmetric colour matching condition.

Logvinenko & Tokunaga instructed their four observers to find a paper lit by one of the six lights that was least dissimilar to the paper lit by the other light [35]. They call the observer’s choice a “match.” The average chromaticity of the matches for a given test Munsell paper taken over four observers and three repetitions is marked in Figs 7 and 8 with a square. The squares have been connected by red lines to make them easier to see as a group. A circular dot in these figures stands for the chromaticity of the test paper under the matching illumination. When a circle and square of the same colour are close together it means that for that test paper an exact match (i.e., perfect colour constancy in traditional terminology) generally took place. When a circle is close to a square of a different colour it means that for that test paper there was generally a mismatch. In other words, the observers chose a different Munsell paper under the matching illumination than the given test paper.

As can be seen in Fig 7 (upper left) and (upper right), for the yellow illuminant the majority of the overlapping circles and squares are of the same colour, and when they do not overlap, they are nonetheless quite close together. The accuracy of the matching under this asymmetric illumination condition was found to be only slightly less than under symmetric illumination conditions, whether neutral-neutral or yellow-yellow. Specifically, the average exact match rates (percentage of cases when the same paper was chosen as the match) for observers for the symmetric illumination conditions neutral-neutral and yellow-yellow were 92% and 93%, respectively. The average exact match rates for the asymmetric illumination conditions NY and YN were 77% and 80%, respectively. Therefore, the matches obtained by Logvinenko & Tokunaga (2011) when one illuminant was neutral and the other yellow can be qualified as rather good (if not perfect) colour constancy by the standards used in other colour constancy studies.

We also computed the centroids of the metamer mismatch volumes and projected them onto the chromaticity diagrams in Figs 7 and 8. We will refer to these projected points as *mismatch centroid chromaticities*. Predictions were also made using the von Kries [39] coefficient-rule-based model of colour constancy. Since the CIE 1931 colour matching functions $\overset{―}{x}(\lambda ),\overset{―}{y}(\lambda ),$ and $\overset{―}{z}(\lambda )$ are used as the *s*_*i*_(*λ*) in Eq (1), the colour signal is represented initially in CIE XYZ coordinates. In order to make predictions of a von Kries type, the XYZ are first transformed to a new set of primaries using the Hunt-Pointer-Estevez transformation as described by Hunt [40, 41]. This transformation is used, for example, as a component of the RLAB colour appearance model [15].

The plots compare the asymmetric colour matches made by observers in Logvinenko & Tokunaga’s experiment to predicted matches based on the mismatch centroid chromaticities and on the von Kries coefficient rule under 4 different illumination conditions. In particular Fig 7 (upper left) shows: (i) the chromaticities of the 20 Munsell stimulus papers (Fig 2 (left)) under the yellow light indicated by circles connected by the black lines; (ii) the averaged matches indicated by squares connected by the red lines; (iii) the mismatch centroid chromaticities for the neutral/yellow (NY) illumination condition indicated by asterisks connected by the green lines; and (iv) the von-Kries-coefficient-rule-based prediction for the neutral/yellow (NY) illumination condition indicated by diamonds connected by the blue lines. Similarly, Fig 7 (upper right) shows the analogous results for the yellow/neutral (YN) illumination condition. Note that all the data in Fig 7 (upper right) are from an entirely separate, though related, experiment than for Fig 7 (upper left). In Fig 7 (upper left) the squares connected by red lines indicate the matches made under the yellow illuminant when the stimulus papers are lit by the neutral illuminant. In Fig 7 (upper right) it is the other way around: matches are made under the neutral illuminant when the stimulus papers are lit by the yellow illuminant. Similarly, in Fig 7 (upper left) the mismatch centroid chromaticities and the von-Kries-based predictions are calculated for the NY condition, while in Fig 7 (upper right) they are calculated for the YN condition.

Fig 7 (upper left) and (upper right) facilitate comparison of the two methods of predicting matches relative to the actual observer matches. It is evident from the figures that for the NY and YN illumination conditions the observers’ matches are generally more accurate than those provided by either of the prediction methods. Still, the predictions, by and large, are not too far from the matches for both the NY and YN cases. However, for other illumination conditions the accuracy of the predictions turns out to be much worse. Nonetheless, the observers’ matches shown in Figs 7 (lower left) through 8 (lower right) are much closer to the stimulus papers than the predictions based on either the mismatch centroid chromaticities or the von Kries coefficient rule. The von Kries predictions are especially bad. In fact, the von Kries coefficient rule even predicts points falling outside the chromaticity diagram (i.e., it predicts non-existent lights). The mismatch centroid chromaticities make reasonable predictions only for the NB and NG illumination conditions.

## Colour constancy or colour alteration?

As the observers’ performance in the Logvinenko & Tokunaga experiment is quite good, it is tempting to attribute the errors in the observers’ matches—which occur even when both illuminants are neutral—to unavoidable human performance variability and measurement error, and conclude that the experimental results do not seriously undermine the idea of colour constancy. That is, perfect colour constancy (i.e., a 100% exact match rate) is quite possible, but hard to achieve under real experimental conditions. However, such a conclusion would be a mistake—perfect colour constancy (other than by chance) is impossible in principle because of metamer mismatching.

**Fig 9:**
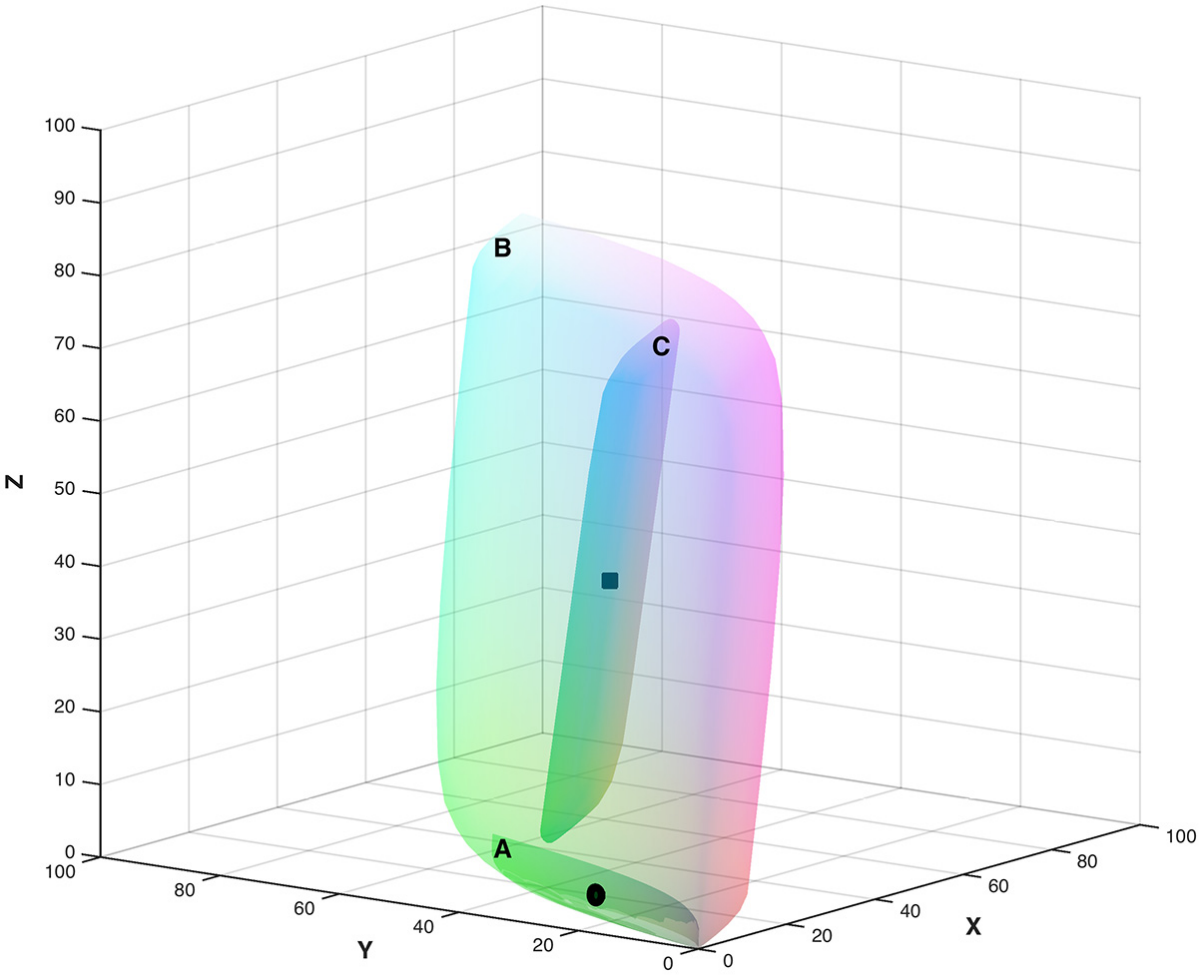
Label A indicates the object-colour solid under green (G) illumination. B indicates the object colour solid under the neutral (N) illumination. The black dot indicates flat grey under green (G). C indicates the metamer mismatch volume of the flat grey for a change in illumination from green (G) to neutral (N). The black square shows flat grey under the neutral (N) illumination.

**Fig 10:**
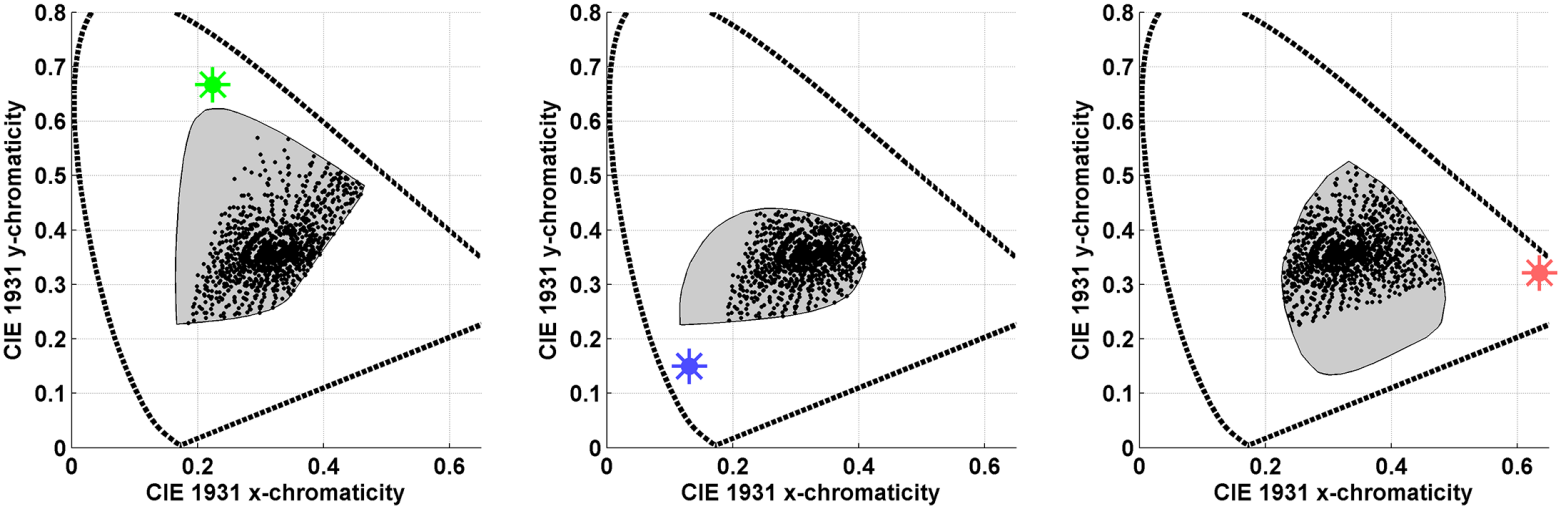
Chromaticity mismatch areas for several illumination conditions. Chromaticity mismatch areas (grey regions) for the following illumination conditions: (Left) green (G) to neutral (N); (Centre) blue (B) to neutral (N); and (Right) red (R) to neutral (N). The green, blue and red asterisks indicate the chromaticity of the flat grey reflectance under the G, B and R illuminants respectively. The black markers are the chromaticities under neutral illumination (N) of the numerous Munsell papers that fall inside each of the metamer mismatch areas.

Consider, for example, the GN illumination condition. Fig 9 shows the object-colour solids for the green and neutral illuminants, and the metamer mismatch volume for the flat grey (i.e., for the object-colour solid centre) inside the latter. It is large with a metamer-mismatch index of 9.7%. The corresponding chromaticity mismatch area is presented in Fig 10 (left). For each point of this metamer mismatch volume there exists a reflecting object such that the CIE XYZ tristimulus coordinate of the light reflected by it under the green illumination is equal to that reflected by the flat grey, while the CIE XYZ tristimulus coordinate of the light reflected by it under the neutral illumination differs from that reflected by the flat grey. Let us call a sample of such objects representing each point in the metamer mismatch volume the *critical sample*. If one assumes that under a single, spatially homogeneous illumination, reflecting objects have identical colour appearance if and only if they are metameric (we will refer to this as the *basic assumption*), then one must admit that all the objects in the critical sample will have a different colour appearance under the neutral light than they have under the green light.

**Fig 11:**
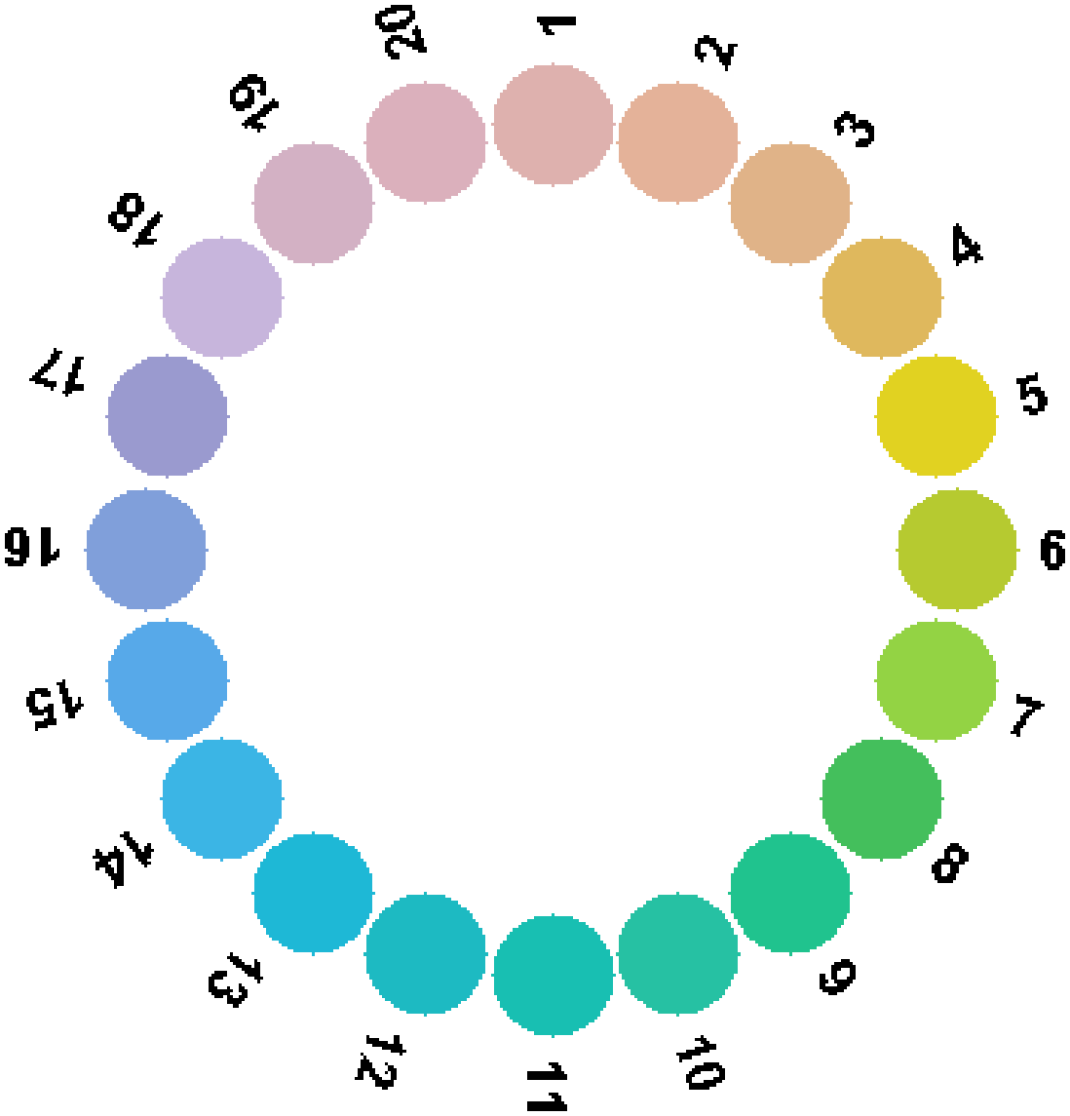
Pictorial representation of the 20 Munsell papers lying inside the metamer mismatch volume of flat grey for the green-to-neutral illumination condition. The colours in the figure only approximate those of the actual Munsell papers. For the correct colours refer to the actual Munsell papers, which starting from 1 are: 5R 8/6, 10R 8/6, 5YR 8/6, 10YR 8/8, 5Y 8.5/12, 10Y 8/10, 5GY 8/10, 10GY 7/10, 5G 7/10, 10G 7/8, 5BG 7/8, 10BG 7/8, 5B 7/8, 10B 7/8, 5PB 7/8, 10PB 7/8, 5P 7/8, 10P 8/6, 5RP 8/6, and 10RP 8/6.

The following question arises: How large is the difference in colour appearance within the critical sample under the neutral light? To answer this question we ascertained which of the color signals from the 1600 papers of the Munsell glossy collection [38] fall into the metamer mismatch volume depicted in Fig 9. It turns out that, being illuminated by the neutral light, 14% of the 1600 Munsell papers reflect light having CIE XYZ tristimulus values that fall into the metamer mismatch volume in question. Given the basic assumption, while this subset of the Munsell collection is, by no means, metameric to the flat grey under the green illumination (thus not included in the critical sample), these Munsell papers represent the colour of some objects from the critical sample under the neutral light. In other words, these Munsell chips give an indication as to the range of colours the flat grey colour might become. As can be seen from Fig 11, this range is very large. This figure renders a series of 20 Munsell papers from every other page of the Munsell Book of Colour that, in fact, all lie inside the metamer mismatch volume for flat grey. Note the high Munsell chroma of these, which is 8 or higher for 14 or the 20 papers.

So, metamer mismatching results in the paradoxical fact that there exists a set of objects having identical colour under one light, but a quite varied set of different colours under another light. In particular, there are 20 reflecting objects that all appear the same as neutral grey under the green illumination but which under the neutral illumination turn into the set of different saturated colours making a hue circle as in Fig 11. This example undermines the very idea of colour constancy understood as the constancy of colours per se. In other words, what can the constancy of colours possibly mean if a single colour like grey can become any of the other colours shown in Fig 11? Any transformation intended to model the change in the colour from one illumination to the other can only be constant for one of the resulting colours and must be inconstant for all the rest.

Indeed, the question of whether the *colour* that the flat grey reflectance appears under the green illumination is constant under a change to the neutral illumination is ill-posed because the answer depends on which *object* is the carrier of this colour. The answer will be affirmative only for those carriers that are metameric to the flat grey (under both the green and neutral lights), assuming also that the flat grey appears achromatic under both the lights, and the answer will be negative for all the others. Hence, the notion of colour constancy can apply only to objects, not to colours. Yet, if colour constancy is understood as the constancy of the colour of a particular object, one has to admit that there is only one object from the critical sample, namely, the one metameric to the flat grey under the neutral illumination, that will exhibit colour constancy. All the remaining sample objects will be inconstant since their colour appearance will be different under the neutral illumination.

It follows that the dichotomy “constancy vs. inconstancy” proves to be restrictive and confusing, if not inadequate. A change of illumination generally affects the colour of an object to a considerable extent. It would be better to describe the effect as the object colour being altered rather than its colour constancy being violated. This is in contrast to the view that object colour is fundamentally constant, subject only to occasional minor perturbations. Fig 11 shows that when the illumination switches from green to neutral, the colours of the objects in the critical sample—which are all the same under the green illumination—alter into a wide range of colours. The metamer mismatch volume bounds the set of altered colours.

Interestingly, the proportion of Munsell papers falling into the chromaticity mismatch area (for the GN illumination condition) is 81%, which is much larger than the proportion falling into the metamer mismatch volume (14%). Fig 10 (left) shows the chromaticity coordinates of the Munsell papers within the chromaticity mismatch area. The papers falling outside the metamer mismatch volume but nonetheless lying inside the chromaticity mismatch area differ from the corresponding objects in the critical sample only in luminance. In other words, the difference in colour appearance between these Munsell papers and the corresponding objects in the critical sample is only that induced by luminance. If one neglects this luminance-induced colour-appearance difference then one can claim that the range of colours into which grey (under the green light) can be altered (under the neutral light) encompasses more than 81% of the Munsell collection. Interestingly, nearly the same percentage was found for the red light (82%). Percentages for the other illumination conditions can be found in Table 2 (see also Fig 10 (centre and right panels)).

**Table 2:** The number and percentage of Munsell papers falling within the metamer mismatch volume (MMV) and chromaticity mismatch areas (CMA) of flat grey for four illumination conditions involving N (neutral).

	Illumination condition			
	GN	BN	YN	RN
MMV	223 (13.9%)	71 (4.4%)	1 (0.001%)	452 (28%)
CMA	1293 (80.8%)	1132 (70.8%)	122 (7.6%)	1311 (81.9%)

The metamer mismatch volume for the object-colour-solid centre is likely to be the largest one [34]. As mentioned above, the metamer mismatch volume for a point on the object-colour-solid boundary degenerates to a singleton. When moving from the object-colour-solid centre towards the object-colour-solid boundary the metamer mismatch volume was found to shrink. Therefore, the range of the illuminant-induced colour alteration will be less for saturated colours, particularly, for Munsell papers with high Munsell chroma. Since the papers used by Logvinenko & Tokunaga (2011) are all of high chroma, one might argue, perhaps, that the range of the altered colours of these papers was so limited that metamer mismatching could not significantly affect the observers’ performance. Furthermore, as the range of the colour alteration caused by the change between the yellow and neutral illuminants is quite small, maybe the notion of colour constancy can be retained for some illuminants, such as the phases of daylight, even if the notion is inadequate in general.

However, this is not the case. Even for the NY illumination condition and the high-chroma papers of the sort used by Logvinenko & Tokunaga we run to the same problem as outlined above. Indeed, consider the chromaticity mismatch areas induced by a change from the neutral to the yellow illuminant (Fig 4) and how they overlap. There are two Munsell papers, #15 and #14, both of which map to the metamer mismatch volume of paper #15. Denote them as objects *x*_1_ and *x*_2_, respectively. Denote also the metamerism of reflectances under the neutral and yellow illuminants as ∼_*N*_ and ∼_*Y*_ respectively. In other words, two reflectances *x* and *y* will be ∼_*N*_-metameric (written: *x* ∼_*N*_ *y*) if the CIE XYZ tristimulus values of the light reflected by them under the neutral illumination are equal. Likewise, *x* ∼_*Y*_ *y* means that the CIE XYZ tristimulus values of the light reflected by them under the yellow illumination are equal. Given metamer mismatching, there exists a reflectance $x_{1}^{\prime }$ metameric to *x*_1_ under the neutral illumination (i.e., $x_{1}{∼}_{N}x_{1}^{\prime }$) that is not metameric to *x*_1_ under the yellow illumination (i.e., $x_{1}{≁}_{Y}x_{1}^{\prime }$).

Assume now that perfect colour constancy exists for objects *x*_1_ and *x*_2_. In fact, in Logvinenko & Tokunaga’s experiment, the asymmetric NY match for paper *x*_1_ was *x*_1_, and for *x*_2_ it was *x*_2_ so this might seem plausible. But can it always be the case? Given perfect colour constancy, the asymmetric match for object $x_{1}^{\prime }$ has to be $x_{1}^{\prime }$. However, this leads to a problem. Two objects—*x*_1_ and $x_{1}^{\prime }$—that have the same colour under the neutral illumination (because $x_{1}\;{∼}_{N}\;x_{1}^{\prime }$) match objects under the yellow illumination that now differ in colour (because $x_{1}{≁}_{Y}x_{1}^{\prime }$). By all standards such observer performance would appear paradoxical. Objects *x*_1_ and $x_{1}^{\prime }$ might either match *x*_1_, $x_{1}^{\prime }$, or some third object; but it makes no sense for two indistinguishable objects to match anything but the same object, whatever it happens to be, under the second illuminant.

One might argue that although an object such as $x_{1}^{\prime }$ exists in principle, its reflectance may be so unnatural that it should be excluded from further consideration. Note, however, that there is nothing particularly special about $x_{1}^{\prime }$. There are infinitely many potential reflectances metameric to *x*_1_ under the neutral light that nonetheless will not be metameric to *x*_1_ under the yellow light. For the paradoxical colour dispersion to occur, $x_{1}^{\prime }$ can be, in fact, any reflectance that under the yellow light maps into the metamer mismatch volume induced by $x_{1}^{\prime }$ except those that are ∼_*Y*_-metameric to *x*_1_. By this line of reasoning, there can be no perfect colour constancy for any of these metameric reflectances. In other words, perfect colour constancy is not possible for any ∼_*N*_-metamer of *x*_1_ with the exception of those that are also ∼_*Y*_-metameric to *x*_1_. For all these objects colour alteration rather than colour constancy will be the result.

The situation becomes even more dramatic when instead of the yellow light we consider the other lights. For instance, for the NR illumination condition the metamer mismatch volume of paper #10 includes the CIE XYZ tristimulus values of 11 papers (#8 to #18). Each of these papers can be taken as object *x*_2_. Let us denote these Munsell papers *x*_1_, …, *x*_11_. Hence, in theory at least, one can find 11 objects (not necessarily Munsell papers) $x_{1}^{\prime },\ldots ,x_{11}^{\prime }$ metameric to *x*_1_ under the neutral light (i.e., for each *i* $x_{1}{∼}_{N}x_{i}^{\prime }$) that will then disperse under the red light into 11 different colours so that for each *i* $x_{i}{∼}_{R}x_{i}^{\prime }$. If perfect colour constancy existed, that is, if for each $x_{i}^{\prime }$ an exact asymmetric match took place, then it would be a fascinating visual phenomenon: by some magic, the observer would be able to match correctly each of the 11 objects that are all of identical colour under the neutral light to its corresponding object under the red light, even though the 11 objects then would have 11 different colours lying on half the hue circle.

**Fig 12:**
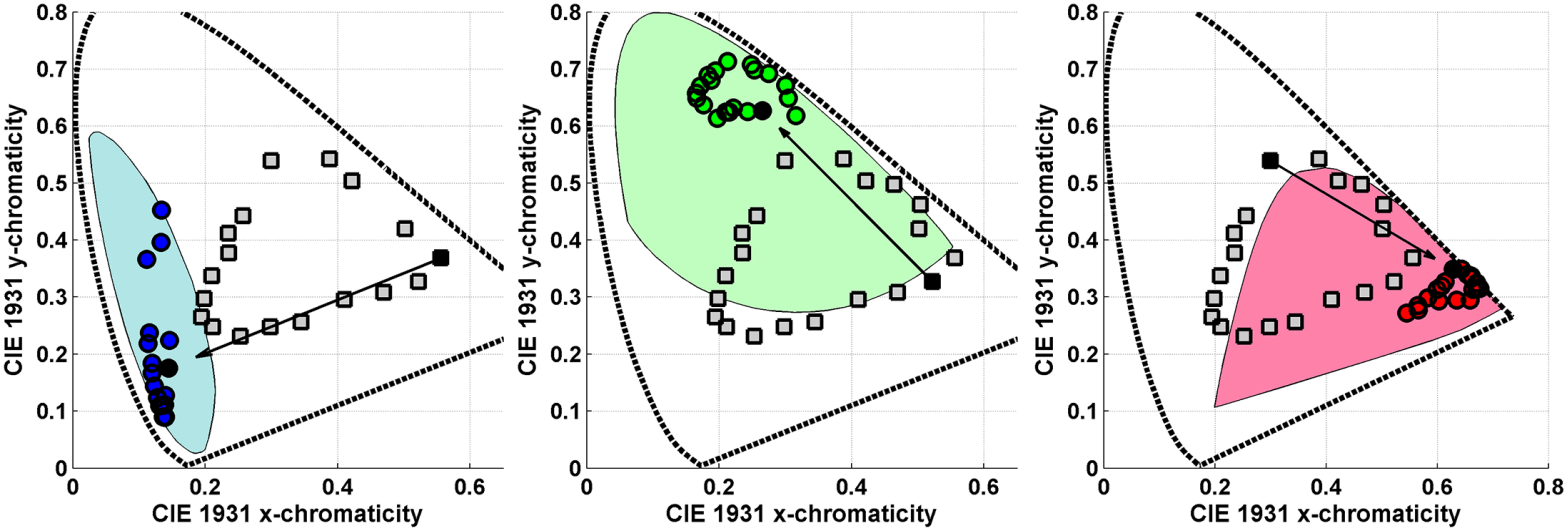
Chromaticity mismatch areas for the neutral-to-blue, neutral-to-green and neutral-to-red illumination conditions. Squares indicate chromaticities of Munsell papers under the neutral (N) illumination. Dots indicate the corresponding chromaticities of the papers under the second illumination. A black square and black dot correspond to the same paper. (Left) Paper #2 under the neutral-to-blue condition. Its chromaticity mismatch area (bluish region) covers the chromaticities of 18 of the 20 Munsell papers. (Centre) Paper #1 under the neutral-to-green condition. Its chromaticity mismatch area (greenish region) covers all 20 Munsell papers. (Right) Paper #8 under the neutral-to-red condition. Its chromaticity mismatch area (reddish region) also covers all 20 Munsell papers.

As pointed out above, metamer mismatch volumes turn out to be rather thin along the luminance axis; nonetheless, the chromaticity mismatch areas can be quite extensive—some of them contain the chromaticities of nearly all 20 Munsell papers. For example, under the blue light, paper #2 disperses into a chromaticity mismatch area that covers 18 of the 20 Munsell papers (Fig 12 (left)). Moreover, paper #1 under the green light disperses into a chromaticity mismatch area covering all 20 Munsell papers (Fig 12 (centre)), as does paper #8 under the red light (Fig 12 (right)). Therefore, the colour dispersion example described above can be further strengthened. One can select 20 objects of identical colour when lit by the neutral light that will be dispersed into 20 different colours lying on a hue circle when lit by the red (or green) light that will differ from those depicted in Fig 2 (left) only in that some colours will have a different luminance factor.

Foster et al. express the opinion that metameric reflectances are rather rare in natural scenes [42]. One might argue that if metamers are rare, then, while theoretically possible, metamer mismatching is very unlikely to be found in the natural world; and thus that the colour dispersion phenomenon described above might have no practical impact, being only of theoretical importance for colour science. It should be kept in mind, however, that metamer mismatching is only the limiting case of a more general phenomenon called the *colour stimulus shift* [36, 43]. As the metamerism of reflectances (defined as metamerism of the lights reflected by the objects) depends on the illumination, reflectances shift from one class of metamerism to another when the illumination changes. As a result, not only is it the case that hitherto metameric reflectances can become not metameric (metamer mismatching) but also reflectances that are close to one another in colour-signal space under one illuminant, can become more separated under some other illuminant. Conversely, reflectances that are far apart in colour-signal space under one illuminant might become close to each other under a different illuminant. Hence, the colour-stimulus shift phenomenon may reveal itself not only in the way it disperses hitherto metameric reflectances into a volume in colour-signal space under a second illuminant (the limiting case of colour stimulus shift), but also in the way it alters the pattern of proximity of reflectances in colour-signal space. It should be emphasized that the relative proximitiy in colour-signal space of all reflectances changes when the illumination changes. In other words, all reflectances undergo an illuminant-induced colour-stimulus shift to some extent.

It follows that the paradox emerging from the colour dispersion described above remains in force even when the requirement of metamerism is dropped. More specifically, one can take into consideration not only the reflectances metameric to some particular object, say, *x*_1_, but also those mapping to the vicinity of *x*_1_ in colour-signal space. In other words, the selection of the critical sample of objects forming a hue circle in the colour signal space under the second illuminant can be made not only from the reflectances metameric to *x*_1_ but from all those reflectances that are relatively close to *x*_1_ in colour-signal space.

More formally, let us generalize a notion of metamer mismatch volume induced by switching from an illuminant *p*_1_ to an illuminant *p*_2_ as follows. Define a *multiple metamer mismatch volume* for an arbitrary neighborhood *A* of an object *x* in the colour signal space under illuminant *p*_1_ as the intersection of the metamer mismatch volumes for each point in *A* induced by switching from *p*_1_ to *p*_2_. Admittedly, as the neighborhood *A* gets larger, the multiple metamer mismatch volume becomes smaller. Nonetheless, it is quite possible that for a chromatic illuminant one can find a relatively small neighborhood *A* such that the corresponding multiple metamer mismatch volume encompasses objects of various colours. Although the reflectances in *A* are not all metameric, their colours will be very similar. In such a situation the paradox will be not that the identical colour disperses into the whole variety of hues, but that very similar colours do.

The advantage of considering the multiple metamer mismatch volume is that, firstly, one does not have to choose metameric reflectances to build a critical sample of objects. Secondly, it also answers the other possible criticism that the spectral reflectance functions of the *m* objects $x_{1}^{\prime },\ldots ,x_{m}^{\prime }$ considered above might happen to be so unusual that they would be unlikely to occur in practice. Freeing the choice of objects form the metamerism requirement means that such criticism loses its force.

## From colour constancy to material colour shift

As argued above, the colour constancy phenomenon needs to be reconsidered, first of all, because the colour appearance of objects obviously does alter as the illumination changes, so simultaneous colour constancy with respect to illumination cannot be taken literally. If colour appearance remained unaltered then we would not be able to perceive when and how illumination changes, which is also ecologically important for survival. If the term “colour constancy” is to be retained, it needs to be redefined, not taken literally. It cannot be defined as the constancy of colour appearance, both because the appearance of object colours does change with the illumination and because metamer mismatching means that strict colour appearance constancy is impossible in theory.

One might argue, however, that although there is an unavoidable change in colour appearance caused by an illumination change (in space and/or in time), it is perceived as a change in lighting rather than a change in material. Loosely speaking, the argument would be that when the illumination changes we see this change as a change in the apparent illumination, the colour of the object remaining unaltered. Such a view implicitly implies the notion of “intrinsic colour” [44, 45] for both objects and lights. In particular, it assumes that colour is an intrinsic feature of an object that remains constant irrespective of the illumination incident on it; and vice versa, there is also illumination colour (to be distinguished from object colour) that is an intrinsic feature of the illumination that does not depend on what objects it falls upon. However, is the separation of colour into completely independent lighting and material components possible?

To explore this issue further, consider the set of all objects (represented by spectral reflectance functions, *x*(*λ*) ∈ 𝓧), and the set of all lights, each light being represented by its spectral power distribution, *p*(*λ*). Let 𝓟 denote the set of all lights. If two object/light pairs, (*x*_1_, *p*_1_) and (*x*_2_, *p*_2_) have identical colour appearance we say that they are *colour equivalent* (written as (*x*_1_, *p*_1_) ≈ (*x*_2_, *p*_2_)). It would appear safe to assume that equality of colour appearance is transitive, thus colour equivalence is an equivalence relation on the set of object/light pairs 𝓧 × 𝓟. The set of object/light pairs in a class of colour equivalence can be said to have the same object colour. Colour equivalence implies metamerism (i.e., equality of the triplets of the tristimulus values for the lights reflected from objects), but not vice versa [36]. In other words, equality of the triplets of tristimulus values for the lights reflected from objects does not guarantee that they will be equal in appearance. There is abundant evidence for this distinction between metamerism and colour equivalence for scenes with multiple illuminants [29, 46–52].

The fact is that colour equivalence cannot be separated into two independent equivalence relations—one defined on the object set 𝓧 and the other on the light set 𝓟. Indeed, let us assume, to the contrary, that there exist equivalence relations ≈_*x*_ on 𝓧 and ≈_*p*_ on 𝓟 such that object/light pairs (*x*_1_, *p*_1_) and (*x*_2_, *p*_2_) are colour equivalent if and only if both the following conditions hold: (i) objects *x*_1_ and *x*_2_ are equivalent in terms of ≈_*x*_, and (ii) lights *p*_1_ and *p*_2_ are equivalent in terms of ≈_*p*_, or, more specifically, for any *x*_1_, *x*_2_ ∈ 𝓧, and *p*_1_, *p*_2_ ∈ 𝓟

$$\begin{array}{c} (x_{1},p_{1})\approx (x_{2},p_{2})⟺(x_{1}\approx_{x}x_{2})\;\text{and}\;(p_{1}\approx_{p}p_{2}). \end{array}$$ (6)

The usual idea is that the equivalence relation ≈_*x*_ can be interpreted as determining the colours of the objects, and the equivalence relation ≈_*p*_ as determining the colours of the lights. In other words, all the objects that are ≈_*x*_-equivalent are assumed to have the same colour. As this colour does not depend on illumination, it is usually called “intrinsic colour”. Note that the notion of intrinsic colour implicitly includes the notion of colour constancy, that is, the independence of the intrinsic colour from the illumination. However, a factorisation of the colour equivalence relation ≈ into the two independent equivalence relations, ≈_*x*_ and ≈_*p*_, as in Eq (6), is impossible because of metamer mismatching. Indeed, Eq (6) entails that colour equivalence (*x*_1_, *p*) ≈ (*x*_2_, *p*) for one *p* implies colour equivalence (*x*_1_, *p*′) ≈ (*x*_2_, *p*′) for any other *p*′. As metamerism follows from colour equivalence, it means that if two objects *x*_1_ and *x*_2_ reflect metameric lights when lit by light *p* they must reflect metameric lights under any other light *p*′ too. However, this is not possible in general because of metamer mismatching. As a result, the traditional interpretation [13, 53] does not hold; namely, it is incorrect to interpret the alteration of colour appearance that occurs as the scene illumination changes as the result of a change in the apparent illumination colour (considered as being a perceptual counterpart of the illuminant independent of the object) coupled with the apparent constancy of the object colour (considered as being a perceptual counterpart of the object independent of the illuminant).

True, the colour appearance of objects under illumination that varies spatially in its spectral composition can be factored into “material” and “lighting” colours in line with our immediate experience [50–52]; however, the material colour [36] arising from such factoring is not determined solely by the object, nor is the lighting colour determined solely by the illumination. Because of the unavoidable light-surface interactions both the material and lighting colours are determined by both the object’s spectral reflectance and the light’s spectral power distribution taken together. In other words, colour is not an independent attribute of an object, but rather colour is an attribute of a pair—object/light. Intrinsic colours not only do not exist, they simply cannot exist.

If intrinsic colours existed, there would be a correspondence following immediately from their existence between classes of colour equivalence for different lights. Indeed, two pairs (*x*,*p*) and (*x*′, *p*′) would be in correspondence whenever *x* and *x*′ had the same intrinsic colour. The fact that intrinsic colours do not exist does not exclude, however, the possibility of there being such a correspondence between colour equivalence classes that can be used to form the basis for a formal definition of material colour. Evidence for such a correspondence is provided by the fact that observers readily accept the task of asymmetric colour matching even though the entire palette of colour appearances alters with the illumination. If there were no such correspondence then observers would be unable to understand or complete the task. However, is observer performance in asymmetric matching experiments using the least-dissimilar criterion systematic, and in particular is asymmetric colour matching transitive? An affirmative answer comes from Logvinenko & Tokunaga’s experiment [35]. Specifically, they found that if object *x*_1_ lit by light *p*_1_ asymmetrically matches object *x*_2_ lit by light *p*_2_, and, in turn, object *x*_2_ lit by light *p*_2_ asymmetrically matches object *x*_3_ lit by light *p*_3_, then object *x*_1_ lit by light *p*_1_ asymmetrically matches object *x*_3_ lit by light *p*_3_. It follows that the set of all object colours (i.e., the set of classes of colour equivalence) is partitioned into equivalence classes defined by the asymmetric-match relation. All the objects within such an equivalence class asymmetrically match each other. The term *material colour* has been put forth to refer to these classes. In other words, all the objects in a class of equivalence (referred to as *material colour equivalence*) are defined as having the same material colour [36].

Consider an analogy with the hue perception of the colours of lights. Lights that differ in their saturation can be experienced as being of the same hue, but this does not mean that the lights match one another completely. They do not match because they differ in saturation even though they share the same hue. Analogously, two different object colours can match each other in terms of their material colour without matching in other respects.

It is a fundamental capability of human colour vision that any object colour experienced by an observer under some illuminant, can be put into correspondence with some object colour under any other illumination, namely, the object colour lying in the same class of material colour equivalence. The term *material colour map* has been suggested for this correspondence [36]. It is the material colour map that reveals itself in asymmetric colour matching. The material colour map is what engenders the idea of colour constancy with respect to illumination.

It must be stressed that this correspondence across illuminants exists between object colours rather than the objects themselves. Because of metamer mismatching the same object can be assigned different material colours under different illuminants. This phenomenon has been called the *material colour shift* induced by illumination change [36]. As one of several examples of this, an object perceived as having a unitary hue under neutral illumination might appear as being of a binary hue under a chromatic illumination. For instance, a Munsell chip perceived as unique yellow under neutral illumination has been found to appear as being greenish-yellow under a chromatic illumination [54]. In other words, an illuminant change generally results in a change in both the material and lighting colours.

From the phenomenon of material colour shift it follows that perfect constancy does not exist for objects even in terms of material colour. However, the notion of constancy of the material colour of an object is generally justified; in contrast to the notion of constancy of colors, as such. A separate issue is, though, that objects happen to be inconstant even as far as their material colours are concerned. The degree of material colour shift shows how inconstant the material colour of an object can be when it is lit differently.

Given the material colour map, the material colour shift for an object can be evaluated provided that its spectral reflectance and the spectral power distributions of the illuminants are known. Identifying the material colour map (i.e., ascertaining which object colours under which illuminants correspond to one another) is an important future task. Accomplishing this task requires a formal representation of object colours. This can be done in terms of a colour atlas, which has been defined as the Cartesian product of a tri-parametric set of objects, 𝓐_*x*_, (referred to as the object-colour atlas) and a tri-parametric set of lights, 𝓐_*p*_, (referred to as the light-colour atlas) such that any pair object/light (*x*, *p*) is colour equivalent to some unique element in 𝓐_*x*_ × 𝓐_*p*_ [36]. In other words, for any pair (*x*, *p*) there exists a unique element from the object-colour atlas, *a*_*x*_ ∈ 𝓐_*x*_, and a unique element from the light-colour atlas, *a*_*p*_ ∈ 𝓐_*p*_, such that (*x*, *p*) is colour equivalent to (*a*_*x*_, *a*_*p*_).

It has been shown that the set of rectangular spectral reflectance functions (the rectangular metamers) can be used as an object-colour atlas [36, 43]. Each rectangular metamer is specified by three descriptors: purity, *α*, spectral bandwidth, *δ*, and central wavelength, *λ*. Likewise, a special set of lights, each of which has a Gaussian spectral power distribution, can be used as a light-colour atlas [36]. Each such light is also specified by three descriptors, *μ*, *σ*, and *k*, the colorimetric correlates of which are close to dominant wavelength, colorimetric purity and luminance. Therefore, each pair object/light (*x*, *p*) can be specified by two triplets of descriptors: *α*, *δ*, and *λ*, and *μ*, *σ*, and *k*, which determine the colour-equivalent pair (*a*_*x*_, *a*_*p*_).

As there is no material colour shift in 𝓐_*x*_ × 𝓐_*p*_, identifying the material colour map amounts to measuring asymmetric colour matching in 𝓐_*x*_ × 𝓐_*p*_. Specifically, let us pick two lights *a*_*p*_ and $a_{p}^{\prime }$ from the light-colour atlas 𝓐_*p*_. Then, for any object *a*_*x*_ ∈ 𝓐_*x*_ lit by *a*_*p*_, there is an object $a_{x}^{\prime }\in A_{x}$ lit by $a_{p}^{\prime }$ that is accepted by an observer as the asymmetric colour match to *a*_*p*_. Thus, for any given pair of illuminants, asymmetric colour matching leads to a three-dimensional (non-linear) map of the tri-parametric object-colour atlas 𝓐_*x*_ onto itself (referred to as the *asymmetric-colour-matching map*).

One can fix one illuminant, say, *a*_*p*_, as the reference illuminant. Letting the other illuminant, $a_{p}^{\prime }$, run over the tri-parametric light-colour atlas 𝓐_*p*_, we get a tri-parametric family of asymmetric-colour-match maps that fully specify the material colour map. Specifically, one can choose the light $a_{p}^{0}$ in the light-colour atlas 𝓐_*p*_ that is metameric to daylight as the reference illuminant. Then for any other illuminant *a*_*p*_ in the light-colour atlas 𝓐_*p*_, and for any object *a*_*x*_ ∈ 𝓐_*x*_ lit by the light *a*_*p*_, there is an object $a_{x}^{\prime }\in A_{x}$ lit by the reference light $a_{p}^{0}$ such that pairs (*a*_*x*_, *a*_*p*_) and $\left(a_{x}^{\prime },a_{p}^{0}\right)$ are in the same class of material colour equivalence. Operationally, this means that object $a_{x}^{\prime }$ is the asymmetric match to object *a*_*x*_. Let *α*, *δ*, and *λ* be the descriptors of the match $a_{x}^{\prime }$. As these, generally, depend on object *a*_*x*_ and vary with the illuminant *a*_*p*_, we denote them as *α*(*a*_*x*_; *a*_*p*_), *δ*(*a*_*x*_; *a*_*p*_), and *λ*(*a*_*x*_; *a*_*p*_). Note that an important difference between the family of asymmetric-colour-matching maps and the material colour map is that the former depends on the choice of the object- and light-colour atlases, whereas the latter does not.

Given the descriptors of the illuminant *a*_*p*_ (i.e., *μ*, *σ*, and *k*), the descriptors of the match, $a_{x}^{\prime }$, can be expressed explicitly as functions of the illuminant descriptors: *α*(*a*_*x*_; *μ*, *σ*, *k*), *δ*(*a*_*x*_; *μ*, *σ*, *k*), and *λ*(*a*_*x*_; *μ*, *σ*, *k*). These three functions specify the asymmetric colour match for the object colour represented by the object *a*_*x*_ lit by the light *a*_*p*_. We will refer to these as the *asymmetric-colour-matching functions* and they collectively define the asymmetric-colour-matching map. They fully describe how the “colour” of the object *a*_*x*_ changes with the illumination. Note that they are actually functions of six variables as they depend on both the three descriptors of object *a*_*x*_ and the three descriptors of light *a*_*p*_. In fact, we are dealing with a three-parameter family of 3 × 3 maps 𝓐_*x*_ → 𝓐_*x*_.

The asymmetric-colour-matching functions cannot be derived from a priori principle, but must be determined by experiment. Experimental identification of the asymmetric-colour-matching functions implies a physical implementation of some colour atlas. The broadband light from any traditional tristimulus colorimeter is likely to serve as a reasonable implementation of the light-colour atlas. As to the object-colour atlas it is more difficult to implement because of at least two problems. The first is that creating a particular spectral reflectance profile is either difficult (e.g., Gaussian) or impossible (e.g., rectangular). The second problem is that the object-colour atlas is infinite, although clearly we can only make a finite number of physical reflectance samples. Admittedly, as any experiment can be performed only with a finite number of stimuli, we can restrict ourselves to a finite sample of elements from the colour atlas and, hence, the object-colour atlas as well. Such a finite sample of atlas elements we will call an *atlas sample*.

It is worth mentioning, that the decision to employ a finite sample of elements happens to ease the first problem, since for such a sample the spectral reflectances of its elements become a secondary matter. There are, in principle, just two requirements on the light-colour-atlas sample and object-colour-atlas sample. First, there should be no metameric pairs—object/light—in the resulting colour-atlas sample (i.e., the lights reflected from any two objects from the object-colour-atlas sample lit by any lights from the light-colour-atlas sample should be not metameric). Second, the object-colour-atlas sample should represent the object colours more or less evenly in terms of the subjective difference between them. The widely used Munsell Color Book and the 1950 NCS Standard Colours are likely to meet these requirements unless the tristimulus colorimeter used as the light-colour-atlas sample comprises light sources with unusual spectral power distribution functions that happen to make some Munsell chips reflect metameric lights.

For the asymmetric-colour-matching experiment, two copies of the object-colour-atlas sample are required. One copy is to be illuminated by the reference illuminant (e.g., daylight) and the other by a light from the light-colour-atlas sample. The asymmetric-colour-matching task should be performed for each element in the object-colour-atlas sample, and for each element in the light-colour-atlas sample, in other words, for all pairs. However, experimental identification of the asymmetric-colour-matching functions can be made less daunting if we restrict ourselves to a subset of illuminants, for example the Planckian radiators for which the asymmetric-colour-matching functions become functions of just one variable because their spectral power distributions are a one-parameter family. Given that the finite atlases consist of discrete samples, it is unlikely that an exact match can always be found during asymmetric matching, so the least-dissimilar match criteria [35] might be a preferable to exact matching. In the end, for any element from the object-colour-atlas sample lit by any light from the light-colour-atlas sample, an element of the object-colour-atlas sample lit by the reference light will be established as its asymmetric match. The latter object-light pair represents the material colour of the given object-light pair.

The asymmetric-colour-matching functions once they are established will provide a tool for predicting how the colour of a particular object changes when its illumination changes from one illuminant to another. To make such a prediction requires establishing which object-light pair in the colour-atlas sample matches the particular object-light pair in question. Although performing object-colour matching would usually imply adjusting the illumination, in the case of a given object-light pair to be matched, the search for a colour match can be restricted to the elements of the object-colour-atlas sample illuminated by just one, special-for-each-particular-element light [36]. This follows from the straightforward assumption that an object-colour match (colour equivalence) implies the metamerism of the reflected lights. From this it follows that for each element of the object-colour atlas there is only one light in the light-colour atlas for which a match to the given object-light pair is possible. Therefore, to find an object-colour match requires being able to view independently each element of the object-colour-atlas sample while it is being lit by a particular light (from the light-colour-atlas sample) selected so that the light reflected from each such element is metameric to that reflected from the object to be matched. If there is no such metamer-making element in the light-colour-atlas sample then the element from the object-colour-atlas sample can be eliminated from any further consideration as a potential match. Although this procedure may sound onerous, the only difference between it and that of traditional symmetric object-colour matching is that the latter is undertaken under a single homogeneous illumination; whereas, in what is described here each object-colour-atlas element is lit automatically by a light that depends specifically on the object-light pair to be matched.

An object-colour-atlas sample equipped with an automatically adjustable illumination from a light-colour atlas can be called an object-colorimeter since it allows us to measure object colour as described above. Moreover, if the asymmetric-colour-matching functions have been established for such an object-colorimeter then the material colour can be evaluated for an arbitrary object lit by arbitrary light. The results obtained using two different object-colorimeters can be converted into those of the other if the object-colorimeters are calibrated with respect to one another. Such calibration means determining the colour equivalence relationships between their corresponding elements.

Note that object-colorimetry is more complex than classical colorimetry. Firstly, classical colorimetry concerns the colour matching of lights; whereas, object-colorimetry involves reflecting objects. Secondly, characterizing asymmetric colour matching requires a separate asymmetric-colour-map (i.e., a three-parameter family of triplets of asymmetric-colour-matching functions) for each light in the light-colour atlas; whereas, symmetric colorimetric matching requires only three colour matching functions (e.g., the CIE 1931 colour matching functions) for its full specification. It must be kept in mind, though, that classic colorimetric matching usually ignores the transmittance of the atmosphere. If the atmosphere is taken into account, for each atmospheric condition and associated spectral transmittance, a separate triplet of colour matching functions needs to be determined. Furthermore, if the atmosphere’s spectral transmittance varies across the scene, the situation of asymmetric colour matching of lights arises. In this case each pair of atmospheric spectral transmittances from two different scene locations will require a separate triplet of colour matching functions. Hence, the description of the colorimetric matching of lights is no more parsimonious than that of object colour matching unless some simplifying assumptions (e.g., that atmospheric effects can be ignored) are made.

Given the asymmetric-colour-matching functions, one can evaluate the material colour shift for any object under any light. The degree of material colour shift shows how inconstant the material colour of the object is for that light. Hence, once the asymmetric-colour-matching functions are known they will allow the degree of material inconstancy (thus, constancy) of any object to be determined. In contrast to searching for colour constancy, whether perfect or imperfect, as has been done since Helmholtz, we suggest that the asymmetric-color-matching functions provide a theoretically well-founded alternative to investigate instead.

Collectively the asymmetric-colour-matching maps manifest a correspondence that the visual system imposes between light-object pairs. The correspondence is perceptual in its nature, so it cannot be derived from any physical principle, and neither can the asymmetric-colour-matching functions. They have to be identified by experiment. Without them, all attempts to model human performance at colour matching will fail because of the lack of this important link between the sensory inputs and the perceptual outputs.

## Concluding Remarks

The notion of colour constancy implies, first, a question—What happens to the colour of an object when its illumination alters? And second, an answer to the question—The colour remains constant, or at least that while the colour changes with illumination, these changes are, by and large, insignificant. Note that the question implicitly assumes that the colour of an object is a perceptual attribute of the object. We show that because of metamer mismatching colour cannot be an object attribute in any sense. We argue instead that colour is a perceptual attribute of an object-light pair.

Although the notion of colour constancy contradicts immediate observations and experimental data, it has been retained in the conceptual repertoire of colour science based on the following argument: Granted that the colour appearance of an object changes with the illumination, what we see is a change in the colour of the illumination while the colour of the object remains constant, or at least approximately constant. Once again, this line of reasoning implies the existence of “intrinsic” colours as independent perceptual attributes of, separately, the object and, separately, the illuminant. However, this is a flawed dogma inconsistent with metamer mismatching.

We argue that the notion of colour constancy is an unfortunate conceptualization of a true, though more subtle, phenomenon; namely, that although the object-colour palette changes with illumination, people are able to establish a correspondence between this new object-colour palette and the previous object-colour palette. Indeed, both palettes contain whites, unique yellows, unique reds, and so forth. However, the whites are experienced differently under the different illuminants, as are the unique yellows and unique reds. The fact that people make a correspondence between object-colour palettes is a fundamental property of human colour vision, and it should undergo a thorough investigation. Once the correspondence is established, it can be used to answer the question: What colour will an object appear under some particular illumination? We believe that answering this important practical question has been, after all, the rationale for most of the numerous experiments regarding colour constancy.

## Supporting Information

S1 Appendix Glossary of Terms and Symbols. (PDF)

S1 Dataset 5-Transition Metamer Reflectance Data. (ZIP)
