# Supplementary material for: Rethinking Colour Constancy
Source: PLoS One. 2015 Sep 10;10(9):e0135029. doi: 10.1371/journal.pone.0135029 (PMC4565710; doi:10.1371/journal.pone.0135029)
Supplement: S1 Appendix — (PDF) [file pone.0135029.s001.pdf]

1 S1 Appendix: Glossary of Terms and Symbols

Term	Notation	Definition
light set	$\mathcal{P}$	set of positive spectral power distributions, i.e., functions $p(\lambda)$ of wavelength, integrable on the visible spectrum interval $[\lambda_{\min}, \lambda_{\max}]$ such that $p(\lambda) > 0$ for each λ
object set	$\mathcal{X}$	set of spectral reflectance functions, i.e., functions $x(\lambda)$ of wavelength integrable on $[\lambda_{\min}, \lambda_{\max}]$ and such that $0 \leq x(\lambda) \leq 1$.
object-light set	$\mathcal{X} \times \mathcal{P}$	Cartesian product of the object and light sets, i.e., the set of all object/light pairs (x, p), $x \in \mathcal{X}$ and $p \in \mathcal{P}$.
colour equivalence	$\approx$	equivalence relation on $\mathcal{X} \times \mathcal{P}$ such that when a pair of objects x_1, and x_2 illuminated by lights p_1, and p_2, respectively, are colour equivalent, i.e., $(x_1, p_1) \approx (x_2, p_2)$, these object/light pairs have the same colour appearance (i.e., are completely visually indistinguishable).
object colour		class of colour equivalent object/light pairs
object-colour set	$\mathcal{C} = (\mathcal{X} \times \mathcal{P}) / \approx$	set of the classes of colour equivalent object/light pairs
i^{th} colour mechanism	$\varphi_i = \mathcal{X} \times \mathcal{P} \rightarrow \mathbf{R}^3$	the response of φ_i (e.g., cone photoreceptor) to an object/light pair (x, p) is given by Eq. 1
colour mechanism set	$\Phi = (\varphi_1, \varphi_2, \varphi_3)$	set of colour mechanisms
colour signal	$\Phi_p(x) = (\varphi_1(x, p), \varphi_2(x, p), \varphi_3(x, p))$	vector of the colour mechanism responses to $x(\lambda)$ illuminated by $p(\lambda)$
metamerism	$\sim$	equivalence relation on $\mathcal{X} \times \mathcal{P}$ defined as $(x_1, p_1) \sim (x_2, p_2) \Leftrightarrow \Phi_{p_1}(x_1) = \Phi_{p_2}(x_2)$
object-colour solid under illuminant $p(\lambda)$	$\Phi_p(\mathcal{X})$	set of colour signals produced by object set under illuminant $p(\lambda)$
metamer mismatching correspondence	ρ	correspondence between object-colour solids $\Phi_{p_1}(\mathcal{X})$ and $\Phi_{p_2}(\mathcal{X})$ that associates with one another the points in these two object-colour solids that are produced by a common spectral reflectance function
metamer mismatch volume	$\rho(z; p_1, p_2)$	given z in Φ_p, set of points in $\Phi_{p_2}(\mathcal{X})$ with which z is in ρ-correspondence (see Eq. 2)

Term	Notation	Definition
colour atlas	$\mathcal{A}$	a subset of the object-light set $\mathcal{X} \times \mathcal{P}$ that is in one-to-one correspondence with the object-colour set $(\mathcal{X} \times \mathcal{P}) / \approx$.
object-colour atlas	$\mathcal{A}_x$	a subset $\mathcal{A}_x$ in the object set $\mathcal{X}$ such that there is a subset $\mathcal{A}_p$ in the light set $\mathcal{P}$ (a light-colour atlas) such that $\mathcal{A}_x \times \mathcal{A}_p$ makes a colour atlas
light-colour atlas	$\mathcal{A}_p$	a subset $\mathcal{A}_p$ in the light set $\mathcal{P}$ such that there is a subset $\mathcal{A}_x$ in the object set $\mathcal{X}$ (an object-colour atlas) such that $\mathcal{A}_x \times \mathcal{A}_p$ makes a colour atlas
material-colour equiv-alence	$\backsim_m$	an equivalence relation on the object-colour set $\mathcal{C}$ such that the quotient set $\mathcal{C}/\backsim_m$ (i.e., the set of $\backsim_m$equivalence classes) is in a one-to-one correspondence with the object-colour atlas $\mathcal{A}_x$
material colour		class of material-colour-equivalent object-colours
material-colour set	$\mathcal{C}_m = \mathcal{C}/\backsim_m$	set of the classes of material-colour-equivalent object-colours
material colour map	$M : (\mathcal{X} \times \mathcal{P}) \to \mathcal{C}_m$	map assigning to each object/light pair a material colour
asymmetric object-colour match		two object/light pairs (x_1, p_1) and (x_2, p_2) will make an asymmetric (i.e., across-illuminant) colour match if and only if $M(x_1, p_1) = M(x_2, p_2)$.
atlas sample		a finite sample of elements from an atlas (e.g., an object-colour atlas sample is a finite sample of elements from an object-colour atlas)
